# Structural analysis of PTPN21 reveals a dominant-negative effect of the FERM domain on its phosphatase activity

**DOI:** 10.1126/sciadv.adi7404

**Published:** 2024-02-28

**Authors:** Lu Chen, Zijun Qian, Yuyuan Zheng, Jie Zhang, Jie Sun, Chun Zhou, Haowen Xiao

**Affiliations:** ^1^ Department of Pathology of Sir Run Run Shaw Hospital, Zhejiang University School of Medicine, Hangzhou, Zhejiang 310016, China.; ^2^ School of Public Health, Zhejiang University School of Medicine, Hangzhou, Zhejiang 310058, China.; ^3^ Department of Hematology of Sir Run Run Shaw Hospital, Zhejiang University School of Medicine, Hangzhou, Zhejiang 310016, China.; ^4^ Department of Cell Biology, Zhejiang University School of Medicine, Hangzhou, Zhejiang 310058, China.

## Abstract

PTPN21 belongs to the four-point-one, ezrin, radixin, moesin (FERM) domain–containing protein tyrosine phosphatases (PTP) and plays important roles in cytoskeleton-associated cellular processes like cell adhesion, motility, and cargo transport. Because of the presence of a WPE loop instead of a WPD loop in the phosphatase domain, it is often considered to lack phosphatase activity. However, many of PTPN21’s biological functions require its catalytic activity. To reconcile these findings, we have determined the structures of individual PTPN21 FERM, PTP domains, and a complex between FERM-PTP. Combined with biochemical analysis, we have found that PTPN21 PTP is weakly active and is autoinhibited by association with its FERM domain. Disruption of FERM-PTP interaction results in enhanced ERK activation. The oncogenic HPV18 E7 protein binds to PTP at the same location as PTPN21 FERM, indicating that it may act by displacing the FERM domain from PTP. Our results provide mechanistic insight into PTPN21 and benefit functional studies of PTPN21-mediated processes.

## Teaser

PTPN21 is autoinhibited by FERM-PTP association, which could be disrupted by HPV E7.

## INTRODUCTION

**Fig. 1.**
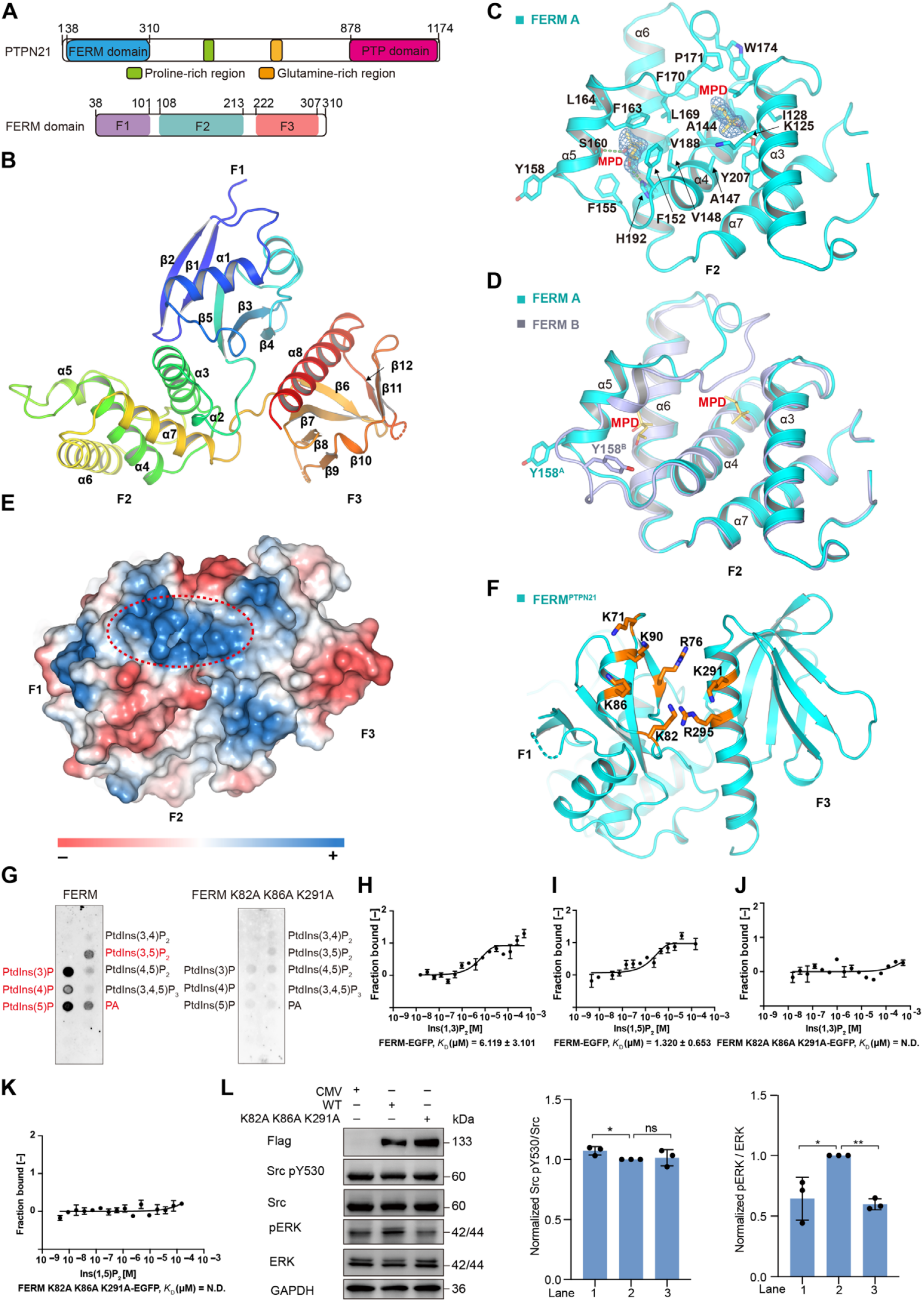
Crystal structure of the FERM domain. (**A**) Diagram of PTPN21 domain organization. The three subdomains (lobes) in the FERM domain are also shown. (**B**) Ribbon illustration of PTPN21 FERM domain structure colored blue to red from N to C terminus. (**C**) Ribbon illustration for the FERM F2 lobe with two MPD molecules bound. The composite omit map contoured at 1.0 σ for the MPD molecules is shown as blue mesh. Hydrophobic residues surrounding the MPD molecules are displayed as sticks. Green dashed lines indicate hydrogen bonds. (**D**) Superposition of the two FERM molecules in the asymmetric unit. FERM A with MPD bound is colored in cyan, and FERM B without MPD is light blue. The region around Y158 adopts different conformation. (**E**) Surface electrostatic potentials of the FERM domain; blue indicates positive potentials, and red indicates negative potentials. (**F**) Residues of FERM^PTPN21^ (orange) potentially involved in binding inositol phosphates. (**G**) Lipid dot-blot assays showed that FERM could bind phosphatidylinositol (PtdIns) mono- or bisphosphates and phosphatidic acid (PA), colored in red. (**H** to **K**) MST titration results of FERM and the FERM K82A K86A K291A triple mutant with Ins(1,3)P_2_ and Ins(1,5)P_2_. ND, nondetected. Data represent mean ± SEM from *n* = 3 independent experiments. (**L**) PTPN21 K82A K86A K291A mutant resulted in reduced ERK phosphorylation in HEK293T cells. CMV: empty vector, WT, or mutant PTPN21 was N-terminal Flag-tagged. For quantitative analysis, each lane of Western blots was sequentially shown as lanes 1 to 3 from left to right. The relative Src pY530/Src and pERK/ERK values were calculated by normalizing to PTPN21 WT. Error bars represent ± SEM of *n* = 3 independent experiments. Statistical analysis was performed using Student’s t test [**P < 0.01, *P < 0.05, not significant (ns) *P* > 0.05].

Protein tyrosine phosphatase nonreceptor type 21 (PTPN21), also known as protein-tyrosine phosphatase D1, is a widely expressed protein tyrosine phosphatase localized in the cytoplasm and plasma membrane (*1*–*7*). It is a protein of 1174 amino acids containing an N-terminal four-point-one, ezrin, radixin, moesin (FERM) domain and a C-terminal phosphatase domain (PTP) (Fig. 1A and fig. S1). An intervening sequence of about 580 residues without apparent ternary structure separates the FERM and the PTP domains (Fig. 1A and fig. S1). The FERM domain is often found within a family of peripheral membrane proteins that link the cytoskeleton to the plasma membrane. It has been proposed that PTPN21 forms a stable complex with actin, Src tyrosine kinase, and focal adhesion kinase (FAK) and localizes at focal adhesion sites, exerting major effects on cell adhesion and migration (*5*). PTPN21 lacking FERM domain does not bind actin and Src, resulting in substantially reduced cell motility (*5*). PTPN21 also directs epidermal growth factor/Src signaling to the nucleus, activating ERK1/2 and Elk1-dependent gene transcription (*3*). The FERM domain of PTPN21 is recently found to interact with the stalk region of the kinesin family member 1C (KIF1C) and stimulates dense core vesicle transport in primary hippocampal neurons, a function that does not require its phosphatase activity (*8*, *9*).

The phosphatase activity of PTPN21 PTP domain is controversial, purified recombination PTPN21 PTP domain was reported to be inactive toward a panel of synthetic phosphopeptides in vitro (*10*, *11*). A catalytically important WPD (Trp-Pro-Asp) loop is present in all members of the FERM-containing PTP subfamily but not in PTPN21, which has a WPE loop. Thus, PTPN21 is often considered a pseduophosphatase (*10*, *11*). However, accumulating evidence has demonstrated that PTPN21 might have weak activity toward certain substrates. PTPN21 was reported to dephosphorylate Src pY530 in human embryonic kidney (HEK) 293 cells (*3*, *5*, *12*). Moreover, PTPN21 is highly expressed in tumor samples from bladder carcinoma, and a catalytically inactive mutation of PTPN21 was shown to inhibit the growth and migration of bladder cancer cells (*6*). In addition, mutations in PTPN21 have also been identified in several human cancers such as colorectal tumors, colon cancer, endometrial cancers, and acute lymphoblastic leukemia (*13*–*19*). Recently, PTPN21 has been shown to be pivotal in maintaining cell mechanical properties and helps retain hematopoietic stem cells (HSCs) in the bone marrow niche, and deletion of PTPN21 decreases cell stiffness and increases mobility and deformability in HSCs, which results in HSCs egress into the peripheral blood (*18*, *19*). This function of PTPN21 specifically involves the catalytic activity of PTPN21 and the dephosphorylation of the cytoskeleton-associated protein Septin1 (*18*). These findings suggest that more work is required to reconcile the contradicting results from previous studies.

To understand the mechanism of action and regulation of PTPN21, we have determined structures of FERM^PTPN21^, PTP^PTPN21^, and PTP^PTPN21^-Src pY530 peptide complex as well as a complex structure of the PTP and FERM domains. Combined with phosphatase assays and site direct mutagenesis, we have demonstrated that PTPN21 PTP is autoinhibited by the FERM domain. Disruption of FERM-PTP interaction results in enhanced downstream ERK activation. We have also revealed that the oncogenic HPV18 E7 (18E7) protein binds to PTP at the same location as FERM but with a much higher affinity, indicating that it may displace the FERM domain from PTP. Unexpectedly, 18E7 binding to PTPN21 notably increased the protein level of 18E7 in cells. These results suggest that 18E7 may exploit PTPN21 in multiple ways.

## RESULTS

### Structure of the FERM domain

The N terminus FERM domain of PTPN21 (18 to 319) crystallized in space group P212121 with two molecules in the asymmetric unit, and the structure was determined at 2.1 Å (Fig. 1, A and B, and table S1). The two molecules are quite similar and adopt the three-lobed cloverleaf architecture (F1, F2, and F3) characteristic of the archetypical FERM domains found in radixin, moesin, and focal adhesion kinase (*20*–*22*). The F1 lobe (21 to 106) exhibits a ubiquitin-like fold and is composed of a five-stranded β sheet covered by two α helices (Fig. 1B). The F2 lobe (107 to 216) contains a core four-helix bundle that resembles acyl-CoA binding protein (*23*–*25*). The F3 lobe (219 to 308) consists of an antiparallel seven-stranded β sandwich with a C-terminal α helix (α8) in between, similar to the pleckstrin homology superfold. The F2 and F3 lobes both interact with F1, and this arrangement holds the three subdomains together (Fig. 1B). The F2 lobe of one of the FERM molecules (molecule A) has two 2-methyl-2,4-pentanediol molecules (MPD; from the crystallization solution) bound (Fig. 1C and fig. S2A). The MPD molecules are located in two hydrophobic pockets separated by L169, F170, P171, and W174 from the loop between α5 and α6 (Fig. 1C and fig. S2A). The MPD binding sites differ from the reported EPB50 binding site on moesin FERM (fig. S2B) (*26*). Because of the presence of MPD, α5 and the surrounding loops display different local structures compared to the other FERM molecule without MPD (Fig. 1D). This in turn results in two kinds of contact interfaces between the two FERM molecules (fig. S2, C to G). From these observations, it appears that the Y158 region has the potential to adopt different conformations and interact with the other FERM domain in distinct ways.

As demonstrated previously, FERM domains could interact with phosphatidylinositol phosphates and promote protein-membrane association (*20*, *22*, *27*, *28*). PTPN21 is found at the plasma membrane and endosomes (*3*, *5*–*7*). Superposition of PTPN21 FERM with inositol-(1,4,5)-trisphosphate–bound radixin FERM structure (*20*) showed that a positively charged groove between F1 and F3 lobe could be the phosphatidylinositol phosphate binding site of PTPN21 FERM (Fig. 1, E and F, and fig. S2H). Using PIP-Strips lipid dot blot analysis, we found that PTPN21 FERM associated with certain phosphatidylinositol phosphate species as well as phosphatidic acid (Fig. 1G). Binding to phosphatidylinositol-3-monophosphate and Phosphatidylinositol-5-monophosphate was reproducibly more than other phosphatidyl mono, di, or tri-phosphates. Triple mutation of positively charged residues (K82, K86, and K291) greatly weakened the FERM-lipid interaction (Fig. 1G). Results from microscale thermophoresis (MST) titration experiments confirmed that PTPN21 FERM indeed interacts with these lipids’ headgroups, such as inositol (1,3) bisphosphate [ins(1,3)P_2_] [dissociation constant (*K*_D_) ≈ 6.11 μM; Fig. 1H] and inositol (1,5) bisphosphate [ins(1,5)P_2_] (*K*_D_ ≈ 1.32 μM; Fig. 1I). Mutation of the positively charged K82, K86, and K291 residues in PTPN21 FERM domain greatly weakened FERM’s affinity for ins(1,3)P_2_ or ins(1,5)P_2_ (Fig. 1, J and K). In HEK293T cells, overexpression of wild-type (WT) PTPN21 led to ERK activation, while the K82/86/291 mutation did not (Fig. 1L).

### Phosphatase activity of PTPN21 PTP domain

**Fig. 2.**
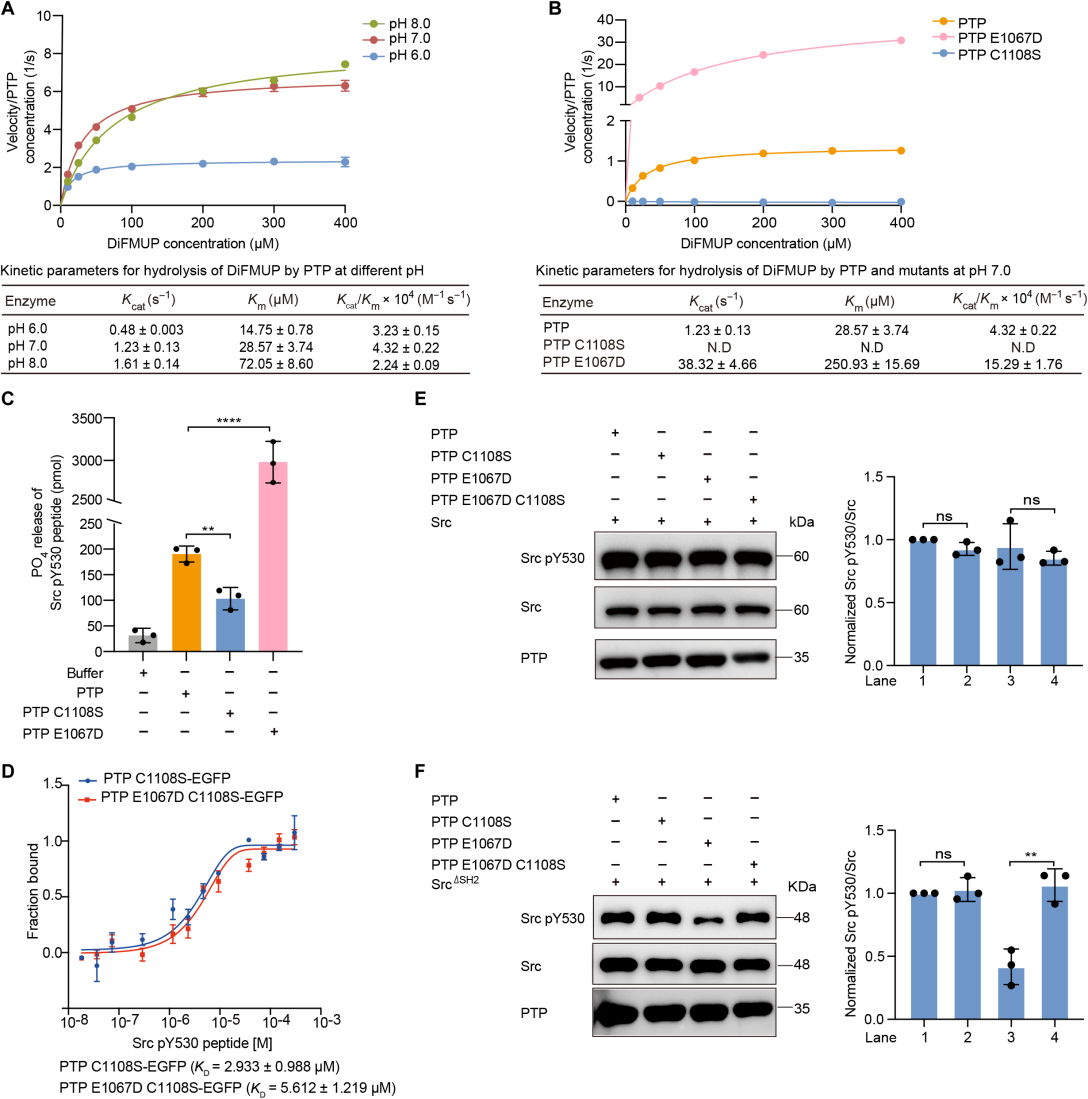
Phosphatase activity of the PTP domain. (**A**) Michaelis-Menten plots of initial rate versus substrate (DiFMUP) concentration using 5 nM PTP in buffers with different pH. (**B**) Michaelis-Menten plots of initial rate versus substrate (DiFMUP) concentration using 5 nM PTP, PTP C1108S, or 0.2 nM PTP E1067D at pH 7.0. Kinetic parameters of PTP C1108S could not be detected (N.D.) as it is inactive. (**C**) Phosphatase activity of PTP, PTP C1108S, and PTP E1067D using Src pY530 peptide (STEPQpYQPGENL) as a substrate, the released phosphate was detected by malachite reagents. The assays were performed by incubating 2.68 μM protein with 268 μM Src pY530 peptide for 30 min at 30°C. (**D**) MST binding affinity measurement of Src pY530 peptide with PTP C1108S (blue) or PTP E1067D C1108S (red). Fluorescent PTP C1108S-EGFP or PTP E1067D C1108S-EGFP (80 nM) was mixed with an increasing amount of Src pY530 peptide. (**E** and **F**) Immunoblot analysis of Src (E) or Src^ΔSH2^ mutant (F) dephosphorylation by PTP, PTP C1108S, PTP E1067D, and PTP E1067D C1108S. Src or Src^ΔSH2^ (0.8 μM) was incubated with 8 nM enzyme for 1 hour at 30°C. N-terminal Flag-tagged Src and Src^ΔSH2^ mutant were expressed in HEK293T cells and purified by Flag-affinity pulldown. For quantitative analysis, each lane of Western blots was sequentially shown as lane 1, lane 2, lane 3, and lane 4 from left to right. For all panels, error bars represent ± SEM of *n* = 3 independent experiments. Statistical analysis was performed using Student’s *t* test. (****P < 0.0001, **P < 0.01, ns *P* > 0.05).

Previous studies showed that PTPN21 could dephosphorylate Src pY530 in HEK293T cells and dephosphorylate Septin1 Y246 in HSC (*3*, *18*). However, in vitro phosphatase assays of PTPN21 PTP against a panel of phosphopeptides showed that PTPN21 PTP was inactive due to the presence of a glutamate residue (E1067) instead of an aspartate residue (D1067) in the catalytically important WPD loop (*10*, *11*). The aspartate residue in most PTPs acts as the general acid to protonate the leaving group during the formation of a phosphocysteine intermediate (*29*–*36*). E1067D mutation of PTP^PTPN21^ exhibited a significantly higher level of phosphatase activity toward *p*NPP or the Eps15^846–854^ peptide compared to its WT counterpart (*11*). To further investigate this matter, we first carried out phosphatase assays using 6,8-difluoro-4-methylumbelliferyl phosphate (DiFMUP) as a substrate. WT PTP^PTPN21^ displayed weak but detectable activity with a *K*_cat_ around 1.2/s at pH 7.0 (Fig. 2A and fig. S3A), about 14 times slower than the well-studied PTP1B (*K*_cat_ around 16.8/s) (*37*). The *K*_m_ for PTP^PTPN21^ toward DiFMUP is around 28.6 μM, 40% higher than PTP1B which is around 20 μM (*37*). Unlike PTP1B, which has an optimal pH around 6.0, PTPN21 is more active in hydrolyzing both DiFMUP and *p*NPP at pH 8.0 (Fig. 2A and fig. S3B), despite its overall lower activity. The PTP^PTPN21^ E1067D mutant is much more active with a *K*_cat_ around 38.3/s, while the active site C1108S mutant is inactive (Fig. 2B). Using Src pY530 synthetic peptide as a substrate, we found that PTP^PTPN21^ resulted in very small amount of phosphate release, while PTP^PTPN21^ E1067D caused 15 times more phosphate release (Fig. 2C). With MST, we found that PTP^PTPN21^ C1108S and PTP^PTPN21^ C1108S E1067D were able to bind Src pY530 synthetic peptide with similar affinities (Fig. 2D). So the observed increase of phosphate release is due to the increased catalytic activity of E1067D. We also purified full-length Src and Src^ΔSH2^ proteins from transiently transfected HEK293T cells as substrates for PTP^PTPN21^. The phosphorylated Y530 in the C terminus tail of Src is normally bound to its SH2 domain, which results in an autoinhibited conformation (*38*, *39*). PTP^PTPN21^ failed to dephosphorylate either Src or Src^ΔSH2^, and PTP^PTPN21^ E1067D was able to dephosphorylate Src^ΔSH2^ but not the full-length Src (Fig. 2, E and F). In HEK293T cells, similar to the results reported by Cardone *et al.* (*3*), overexpression of PTPN21 resulted in a slight reduction of Src pY530 phosphorylation; however, the difference between PTPN21 WT and C1108S was very small (fig. S3C). Together, it appears that PTP^PTPN21^ has weak phosphatase activity toward certain substrates.

### Structure of the phosphatase domain

**Fig. 3.**
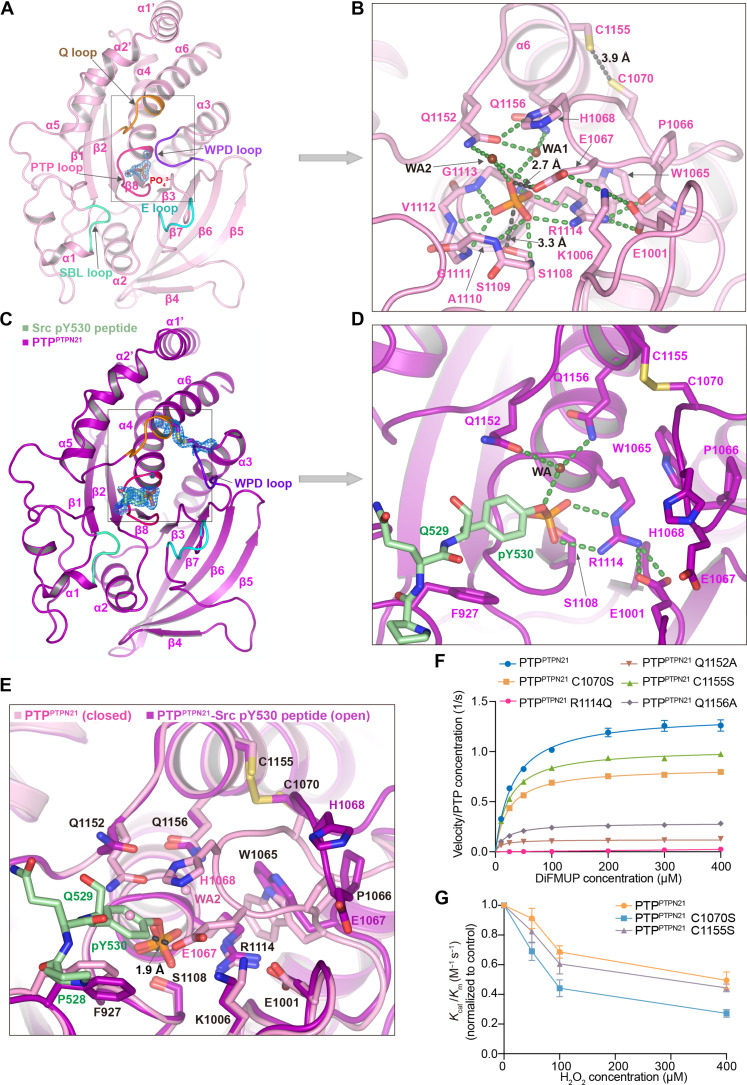
Crystal structure of the PTP^PTPN21^ domain. (**A**) Ribbon diagram showing the overall structure of the PTP^PTPN21^ domain (pink). The PTP loop is colored in red, the WPD loop in purple, the Q loop in orange, the substrate binding loop (SBL) in light green, and the E loop in cyan. The blue mesh is the composite omit map contoured at 2.0 σ for the bound phosphate ion and two catalysis-related water molecules (WA1 and WA2). (**B**) Close-up view of the residues coordinating the phosphate ion and water molecules. The residues involved in the interactions are shown as sticks. Hydrogen bonds and salt bridges are indicated as green dashed lines and the black dashed lines denote the distance between two atoms. (**C**) Ribbon diagram showing the structure of PTP^PTPN21^ (magenta) with Src pY530 peptide (STEPQpYQPGENL, green). The blue mesh is the composite omit map contoured at 2.0 σ for pY530 and an intramolecular disulfide between Cys1070 and Cys1155. (**D**) Expanded view of the area boxed in (C). A bound water molecule (WA) is also shown. (**E**) Superposition of PTP^PTPN21^ (pink) and PTP-Src pY530 peptide complex (magenta). The residues with similar positions in both structures are labeled in black, while the different ones are labeled in their own color. The distance between E1067 from the WPD loop closed PTP structure with pY530 from the PTP-peptide complex is 1.9 Å. (**F**) Michaelis-Menten plots of initial rate versus substrate (DiFMUP) concentration using 5 nM WT or mutant PTP^PTPN21^. (**G**) Relative activity of 5 nM PTP^PTPN21^, PTP^PTPN21^ C1070S, or PTP^PTPN21^ C1155S toward DiFMUP after treatment with increasing concentrations of H_2_O_2_ (0, 50, 100, and 400 μM). The relative *K*_cat_*/K*_m_ values were calculated by normalizing the measured activity to the sample without H_2_O_2_ treatment. For (F) and (G), error bars represent the SEM for at least three independent assays.

To further investigate the working mechanism of the phosphatase domain of PTPN21, we determined structures of PTP^PTPN21^ C1108S in complex with a phosphate ion (Fig. 3, A and B) as well as PTP^PTPN21^ C1108S in complex with a Src pY530 peptide (Fig. 3, C and D). The overall structure of PTP^PTPN21^ C1108S is composed of a twisted central β sheet flanked by α helices on both sides, connected by many loops that make up PTP signature motifs (PTP loop, Q loop, WPD loop, SBL loop, and E loop) (*10*, *11*, *40*, *41*). The structure resembles previously determined classical PTP^PTP1B^ structure [Protein Data Bank (PDB): 1PTV] and the closely-related PTP^PTPN14^ structure (PDB: 2BZL), with root mean square deviations (RMSDs) smaller than 1 Å (fig. S4, A and B) (*30*, *42*).

In the 2.0-Å PTP^PTPN21^ C1108S structure with a phosphate ion in the active site, the electron density of the phosphate ion and two nearby water molecules is well defined (Fig. 3, A and B). The phosphate group is 3.3 Å from the hydroxyl group of S1108, and the oxygen atoms of the phosphate group hydrogen bond to main chain amides in the P loop. The scissile oxygen forms two hydrogen bonds with two water molecules, one of which is the “catalytic water” (WA1) (Fig. 3B). The water molecules themselves are coordinated by Q1152 and Q1156 in the Q loop as well as E1067 in the WPE loop. The carboxyl group of E1067 is close to the scissile oxygen with a distance of 2.7 Å. The WPE loop where E1067 is located is in a closed conformation and aligns well with the closed-WPD loop from PTP^PTP1B^-pY peptide complex (PDB: 1PTV) and HePTP-phosphate complex (PDB: 1ZC0) (fig. S4A) (*30*, *43*). Compared to the PTP^PTP1B^-pY complex, water molecule 2 (WA2) occupies the position of the tyrosine moiety, and Q1152 is in a downward conformation due to the absence of tyrosine. It is likely that WA2 and E1067 as well as WA1 serve to stabilize the phosphocysteine intermediate after P─O bond breakage, and what we captured in Fig. 3B is a product-complex conformation. The invariant R1114 residue is held in place by electrostatic interactions with the inorganic phosphate and E1001. In addition, R1114 forms a hydrogen bond with the backbone carbonyl group of W1065, which helps to maintain the WPD loop in the closed conformation (*36*).

In the 2.0-Å PTP^PTPN21^ C1108S-Src pY530 peptide complex structure, the electron density of the phosphotyrosine residue and the catalytic water is clearly resolved (Fig. 3, C and D). The tyrosine moiety is mainly stabilized by hydrophobic packing with F927, A1110, and V1112, and the phosphate group is coordinated by the P loop and R1114 (Fig. 3D). The catalytic water forms hydrogen bonds with the phenolic oxygen as well as Q1152 and Q1156. The WPE loop is in an open conformation despite the bound peptide. Similar cases have been reported for PTP^SHP-1^-pY peptide complex and PTP^PTP1B^ inhibitor complexes where bound phosphotyrosine or phosphotyrosine mimics did not lead to WPD closure (*44*–*47*). Structural superposition showed that the residues in the open conformation WPE loop of PTP^PTPN21^ C1108S adopt similar positions as their counterparts in PTP^SHP-1^ or PTP^PTPN14^ (fig. S4B). In these structures, neither R1114 of PTPN21 nor R1127 of PTPN14 is close enough to hydrogen bond with the backbone carbonyl group of W1065^PTPN21^/W1077^PTPN14^. In the PTP^SHP-1^-pY structure, the WPD loop is closer to the substrate, and R461 is able to form a weak hydrogen bond with the carbonyl group of W419 with a distance of 3.7 Å, reflecting subtle differences between different phosphatases.

Alignment of the peptide-bound open PTP^PTPN21^ C1108S structure with phosphate-bound closed PTP^PTPN21^ structure showed that the phosphate ion and the phosphate group of the phosphotyrosine are in a nearly identical position, so is R1114, despite around 10-Å shift of the WPE loop (Fig. 3E). Furthermore, E1067 in the closed structure is within steric clash distance (1.9 Å) with the tyrosine moiety in the open structure, further indicating that the closed structure might represent a conformation after P─O bond cleavage. When a phosphotyrosine substrate is present, for catalysis to happen, E1067 would have to move to a certain position between the open and closed conformations currently observed.

Close examination of the electron density revealed that C1155-C1070 in the open structure formed a disulfide bond (Fig. 3, C and D), while C1155 and C1070 in the WPE closed structure are free with a distance around 3.9 Å (Fig. 3B). It is possible that the open conformation promoted C1155-C1070 disulfide bond formation which prevented the WPE loop from clamping down. In vitro under reducing conditions, C1155S or C1070S mutation reduced the *K*_cat_ of PTP^PTPN21^ by around 15 and 25%, respectively, while Q1156A and Q1152A greatly reduced the turnover number of PTP^PTPN21^ (Fig. 3F and fig. S4C). The R1114Q mutant, which is a nontrapping catalytic mutant, was inactive toward DiFMUP and Src pY530 peptide (Fig. 3F and fig. S4, C and D). In MST titrations, PTP^PTPN21^ R1114Q was able to bind Src pY530 peptide with a slightly weaker affinity as PTP^PTPN21^ C1108S (fig. S4E). With increasing concentrations of H_2_O_2_, WT PTP^PTPN21^, C1155S, and C1070S all showed reduced catalytic activity, but C1155S and C1070S were more severely affected at all H_2_O_2_ concentrations tested (Fig. 3G and fig. S4, F to H). Quite a few tyrosine phosphatases like Lymphoid Tyrosine Phosphatase (LYP), SHP2 (PTPN11), Phosphatase and Tensin homolog (PTEN), and Protein Tyrosine Phosphatase Receptor Type U (PTPRU) have back-door cysteines that can protect the catalytic cysteine by forming reversible disulfide bonds (*41*, *48*–*50*). PTPN21 does not have cysteine residues near its active site cysteine C1108. C1155 and C1070 are 13.9 and 13.6 Å apart from C1108, it is very unlikely that they will form a disulfide bond with C1108 as C1155 and C1070 are very close to each other; nonetheless, they appear to offer certain protective effect against oxidation, probably because they are more exposed to solvent.

### Structure of the PTPN21 FERM-PTP^C1108S^ complex

**Fig. 4.**
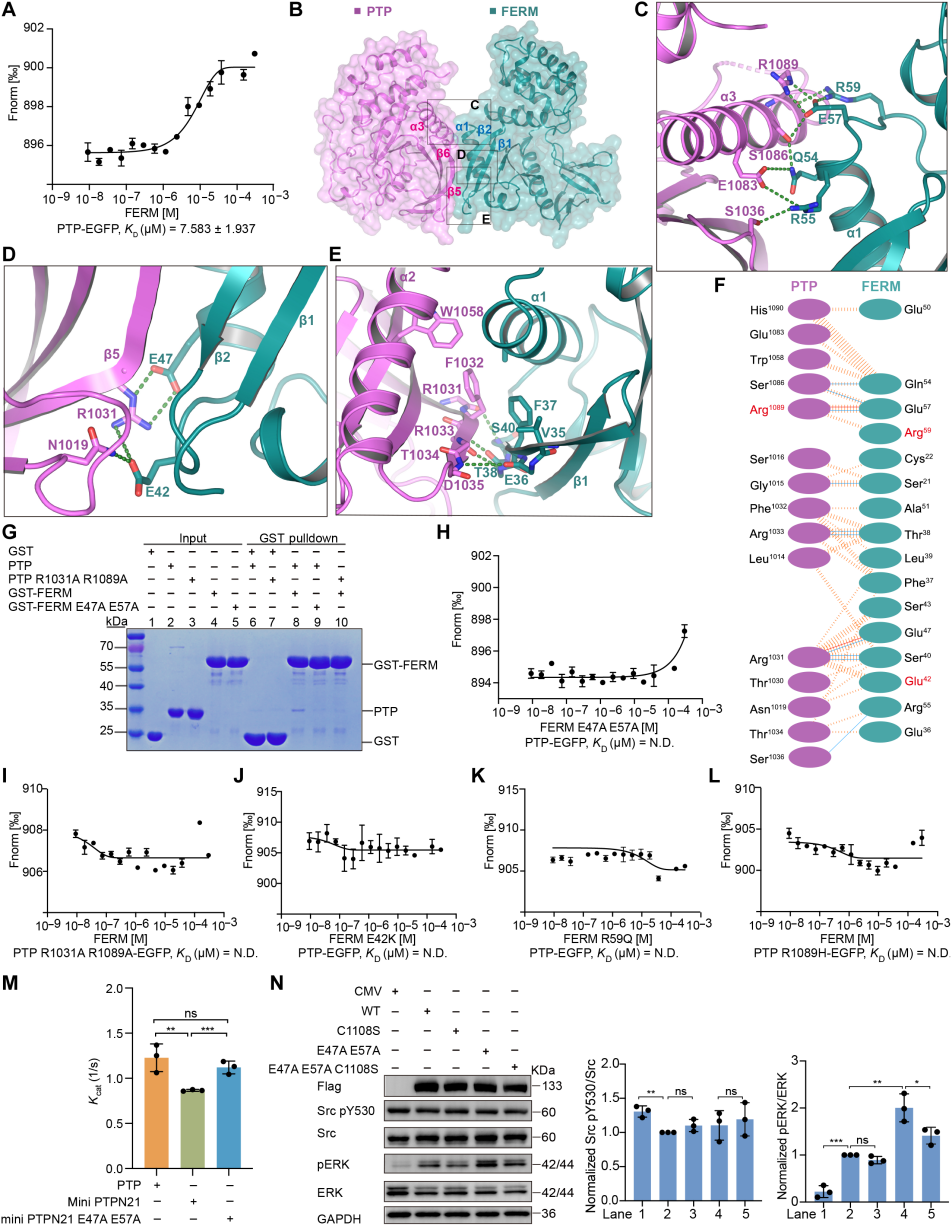
PTPN21 FERM domain interacts with the PTP domain and inhibits PTP activity. (**A**) MST analysis of PTPN21 FERM binding to the PTP domain. (**B**) Ribbon diagram showing the PTP (magenta)-FERM (teal) domain complex (mini PTPN21). (**C** to **E**) Expanded view of the PTP-FERM interaction interfaces boxed in (B). Green dashed lines indicate hydrogen bonds or salt bridges between PTP and FERM domains. (**F**) The residues involved in the PTP-FERM interaction are listed. (**G**) GST pulldown assay showed that the PTP domain could associate with the FERM domain. FERM E47A E57A mutation or PTP R1089A R1031A mutation abolished the binding between the two domains. (**H** and **I**) MST analysis showed that FERM E47A E57A mutation (H) or PTP R1089A R1031A mutation (I) abrogated the interaction between the two domains (*K*_D_ = N.D.). (**J** to **L**) Disease-related mutations of PTP (R1089H) or FERM (E42K and R59Q) abrogated the binding between the FERM and PTP domains as shown by MST analyses. (**M**) The phosphatase activity of mini PTPN21 (FERM-PTP fusion) was significantly lower than PTP or mini PTPN21 with FERM E47A E57A mutation. (**N**) PTPN21 with E47A E57A mutation resulted in more ERK activation in HEK293T cells, WT, or mutant PTPN21 are N-terminal Flag-tagged. CMV, empty vector. For quantitative analysis, each lane of Western blots was sequentially shown as lane 1, lane 2, lane 3, lane 4, and lane 5 from left to right. (A) and (H) to (N) Error bars represent ± SEM of *n* = 3 independent experiments. In (N), the relative values were calculated by normalizing to PTPN21 WT. In (M) and (N), statistical analysis was performed using Student’s *t* test (***P < 0.001, **P < 0.01, *P < 0.05, ns *P* > 0.05). GAPDH, glyceraldehyde phosphate dehydrogenase.

As demonstrated for the FAK, the FAK FERM domain binds to FAK kinase domain and acts as an autoinhibitory module (*51*). We suspect that the FERM domain of PTPN21 might be able to interact with its PTP domain and has a regulatory function. Glutathione *S*-transferase (GST)–tagged FERM domain pulled down a substantial amount of PTP (fig. S5A). MST titration showed that FERM interacted with PTP with a *K*_D_ around 7.6 μM (Fig. 4A). To help crystallize the FERM-PTP complex, we generated a mini PTPN21, replacing the disordered region between the two domains with a flexible linker. After adjusting the linker length, we managed to crystalize the FERM-PTP^C1108S^ complex and determined its structure at 3.0 Å (Fig. 4B and table S1). The mini PTPN21 structure showed that the FERM F1 lobe binds to the α3 and β5 regions of PTP and buries an area around 886 Å^2^ (Fig. 4B). The interaction interface can be further divided into three parts. In the top part, highly conserved residues from FERM α1 region (R59, E57, R55, and Q54) interact with residues from PTP α3 (R1089, S1086, and E1083) through hydrogen bonds and electrostatic interactions (Fig. 4C). In the middle, FERM β2 forms an antiparallel β sheet with β5 from PTP, which is stabilized by main-chain hydrogen bonding and side-chain hydrophobic interactions (Fig. 4D). In the lower part, R1031 from PTP β5 is sandwiched between E42 and E47 from the FERM domain, and E42 also forms a hydrogen bond with N1019 (Fig. 4E). Mutation of critical interface residues in either PTP (R1031A and R1089A) or FERM (E47A and E57A) abolished FERM-PTP association (Fig. 4, G to I). Several mutations found in patients with cancer [E42K, R59Q, and R1089H, from The Cancer Genome Atlas (TCGA) and Catalogue Of Somatic Mutations In Cancer (COSMIC) databases] are located in the FERM-PTP interface (Fig. 4F). MST titrations showed that these mutations greatly weaken the FERM-PTP association (Fig. 4, J to L, and fig. S5, B and C). In vitro phosphatase activity assay showed that the presence of the FERM domain in the mini PTPN21 reduced the PTP domain’s *K*_cat_, while FERM E47A E57A mutant caused less reduction in PTP *K*_cat_, suggesting that FERM binding to PTP negatively affected PTP’s activity (Fig. 4M and fig. S5D). In HEK293T cells, transfection of PTPN21 E47A E57A mutant resulted in more ERK phosphorylation than WT PTPN21, further implying that the PTPN21 is autoinhibited by its FERM domain (Fig. 4N). Moreover, PTPN21 E47A E57A mutant’s activating effect is abolished by the C1108S mutation and R1114Q mutation, indicating that the phosphatase activity of PTPN21 is responsible for the observed ERK activation, despite little observed changes in Src phosphorylation (Fig. 4N and fig. S5E). PTPN21 E42K or R59Q also leads to more ERK phosphorylation in HEK293T, similar to the E47A E57A mutation (fig. S5F).

To investigate whether the binding of inositol phosphate to PTPN21 FERM domain would affect FERM-PTP association, we performed MST analyses of FERM and PTP interaction in the presence of inositol phosphate. The ins(1,3)P_2_ or ins(1,5)P_2_ had no effect on FERM-PTP interaction (fig. S5, G to I). In vitro phosphatase activity assay showed that addition of ins(1,3)P_2_ or ins(1,5)P_2_ to mini PTPN21 and mini PTPN21 E47A E57A resulted in similar *K*_cat_ values as mini PTPN21 alone or mini PTPN21 E47A E57A alone (fig. S5J). So inositol phosphate head groups appear to have little effect on the phosphatase activity of mini PTPN21 in vitro.

The FERM domain in mini PTPN21 adopts a similar overall conformation as molecule B in the FERM alone structure with an RMSD of 0.765 Å over 257 aligned Cα atoms (fig. S5K). The Y158 region in mini PTPN21 is exposed to solvent, and the side chains are poorly ordered. This further underscores the dynamic nature of this region (fig. S5K). The WPE loop in mini PTPN21 is in an open conformation and similar to the WPE loop in the Src pY530 peptide–bound structure (fig. S5L), and the open conformation is in agreement with previous observations that WPD loops favor the open conformation in general (*43*, *52*, *53*). C1155 and C1070 are both free in mini PTPN21 with a distance of 3.9 Å (fig. S5L), and it is possible the upward shift of the R1114 side chain led to a slight rotation of W1065, which in turn resulted in C1070 in a further away position from C1155.

### Competition of HPV18 E7 CR3 with PTPN21 FERM for PTP binding

**Fig. 5.**
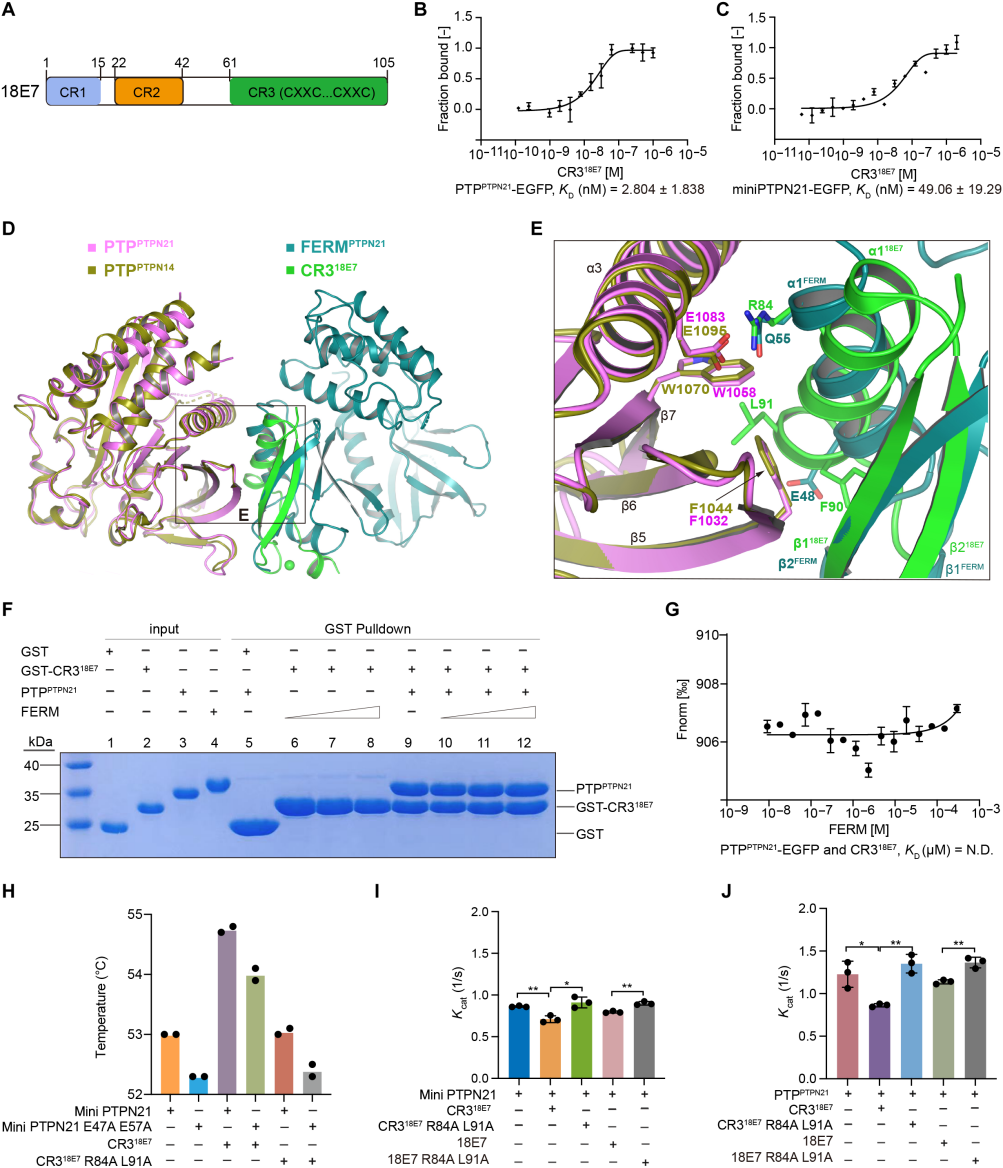
HPV18E7 CR3 competes with PTPN21 FERM for PTP binding. (**A**) Domain architecture of HPV18E7 (18E7). CR, conserved regions. (**B**) MST analysis of CR3^18E7^ binding to PTP^PTPN21^ domain. (**C**) MST titration results of CR3^18E7^ binding to mini PTPN21. Data represent mean ± SEM from *n* = 3 independent experiments. (**D**) Superposition of the PTPN21 FERM (teal)–PTP (magenta) complex with PTP^PTPN14^ (olive)–CR3^18E7^ (green) complex (PDB: 6IWD. (**E**) Close-up view of the boxed area in (D). The residues involved in the interactions are shown as sticks and colored as in (D). (**F**) GST pulldown assay showed that addition of PTPN21 FERM had little effect on the interaction between GST-CR3^18E7^ and PTP^PTPN21^. Equal amount of GST-CR3 and PTP (40 μM) was combined, and then 20 μM (lanes 6 and 10), 40 μM (lanes 7 and 11), or 80 μM (lanes 8 and 12) FERM was added. (**G**) MST analysis showing CR3^18E7^ inhibited FERM^PTPN21^ and PTP^PTPN21^ interaction. CR3^18E7^ (1 μM) was combined with fluorescent PTP-EGFP (80 nM) and then titrated with an increasing concentration of FERM. (**H**) Nano-DSF thermal stability analysis of mini PTPN21 (10 μM) alone or in the presence of 10 μM CR3^18E7^ or CR3^18E7^ R84A L91A mutant. The temperatures of the inflection point for ratio F350/F330 are shown. Data were from two independent experiments. (**I** and **J**) WT CR3^18E7^ or 18E7 reduced the phosphatase activity of mini PTPN21 (I) and PTP (J) toward DiFMUP but the CR3^18E7^ R84A L91A mutant or 18E7 R84A L91A mutant which was defective in PTP binding had little effect. Statistical analysis was performed using Student’s *t* test (**P < 0.01, *P < 0.05).

HPV18 E7 (18E7) is encoded by high-risk alpha papillomaviruses. It is a protein of 105 amino acid residues containing N-terminal conserved region 1 (CR1), CR2 domains, and a C-terminal CR3 domain (Fig. 5A). CR1 and CR2 are relatively flexible and mainly responsible for targeting and promoting the degradation of pocket protein family members, such as retinoblastoma protein (*54*–*56*). CR3 is a zinc finger domain and forms an obligate homodimer, and it regulates multiple viral processes involved in high-risk HPV-mediated tumorigenesis by interacting with several host factors (*57*–*61*).

Several studies showed that the PTP domains of PTPN14 and PTPN21 are able to interact with the CR3 domain of HPV18 viral oncoprotein E7 (CR3^18E7^) (*62*, *63*). In the case of PTPN14, the association of HPV18 E7 caused PTPN14 to undergo proteasomal degradation. Since PTPN14 is a well-known tumor suppressor, degradation of PTPN14 helps to promote the transformation and immortalization of HPV-infected cells (*64*–*66*). However, it appears that PTPN21 protein levels were not affected by the binding of 18E7 in Hela cells, despite high affinities between the two proteins (*63*). MST titrations showed that PTPN21 PTP binds to CR3^18E7^ with an affinity around 2.8 nM (Fig. 5B), nearly two thousand times stronger than the affinity between PTP and FERM. Mini PTPN21 displayed a slightly reduced the affinity for CR3^18E7^ (*K*_D_ ≈ 49 nM; Fig. 5C), indicating that FERM might have some effect on PTP and CR3^18E7^ interaction. Comparison of the PTP^PTPN14^-CR3^18E7^ complex structure (PDB: 6IWD) with our mini PTPN21 structure revealed that CR3^18E7^ and FERM bind to the same location in PTP (Fig. 5, D and E). However, several adaptions render 18E7 protein a tight binder for PTP. First, Q55 in FERM α1 is replaced by R84 in 18E7 α1, which interacts with the negatively charged E1083^PTPN21^/E1095^PTPN14^ in PTP (Fig. 5E). Second, hydrophobic residues in 18E7 α1 (L91 and F90) pack closely with F1032^PTPN21^/F1044^PTPN14^ and W1058^PTPN21^/W1080^PTPN14^ in PTP, while the counterpart of 18E7 L91 and F90 is E48 in FERM (Fig. 5E). As a result of these specific interactions, CR3^18E7^ was able to sequester the binding of FERM to PTP and FERM failed to compete with CR3^18E7^ for PTP binding (Fig. 5, F and G, and fig. S6A). Lee *et al.* (*63*) showed that CR3^18E7^ R84A L91A mutation abolished 18E7 and PTPN21 interaction. With nano- Differential Scanning Fluorimetry (DSF) thermal stability analysis, we found that CR3^18E7^ but not CR3^18E7^ R84A L91A mutant stabilized mini PTPN21 (Fig. 5H and fig. S6B). In addition, CR3^18E7^ and 18E7 but not CR3^18E7^ R84A L91A or 18E7 R84A L91A mutant slightly reduced the DiFMUP phosphatase activity of mini PTPN21 (Fig. 5I and fig. S6C). Similarly, CR3^18E7^ and 18E7 but not CR3^18E7^ R84A L91A or 18E7 R84A L91A mutant reduced the DiFMUP phosphatase activity of PTP alone (Fig. 5J and fig. S6D). Using Src pY530 synthetic peptide as a substrate, we found that CR3^18E7^ have little impact on the phosphatase activity of mini PTPN21 (fig. S6E), while the phosphatase activity of PTP was slightly reduced by the addition of CR3^18E7^ (fig. S6F).

We also looked at whether the binding of inositol phosphate to mini PTPN21 would affect CR3^18E7^ or 18E7 association, and we performed GST pulldown and MST analyses of mini PTPN21 and CR3^18E7^ or 18E7 interaction in the presence of inositol phosphate. The ins(1,3)P_2_ or ins(1,5)P_2_ had minimal impact on mini PTPN21 and CR3^18E7^ or 18E7 interaction (fig. S6, G to L). Moreover, in vitro phosphatase activity assay showed that addition of ins(1,3)P_2_ or ins(1,5)P_2_ to mini PTPN21-CR3^18E7^ or 18E7 did not cause much change in *K*_cat_ values (fig. S6, M to P). Thus, in vitro inositol phosphate head groups appear to have little effect on the interplay between mini PTPN21 and CR3^18E7^ or 18E7. On the other hand, the full length 18E7 displayed weaker affinity (*K*_D_ around 0.2 μM) toward mini PTPN21 when compared to the CR3^18E7^ domain (*K*_D_ around 0.049 μM) (Fig. 5C and fig. S6J), indicating that the N-terminal region of 18E7 somehow interfered with CR3^18E7^ binding to PTP.

### 18E7 interacting with PTPN21 to promote cell migration

**Fig. 6.**
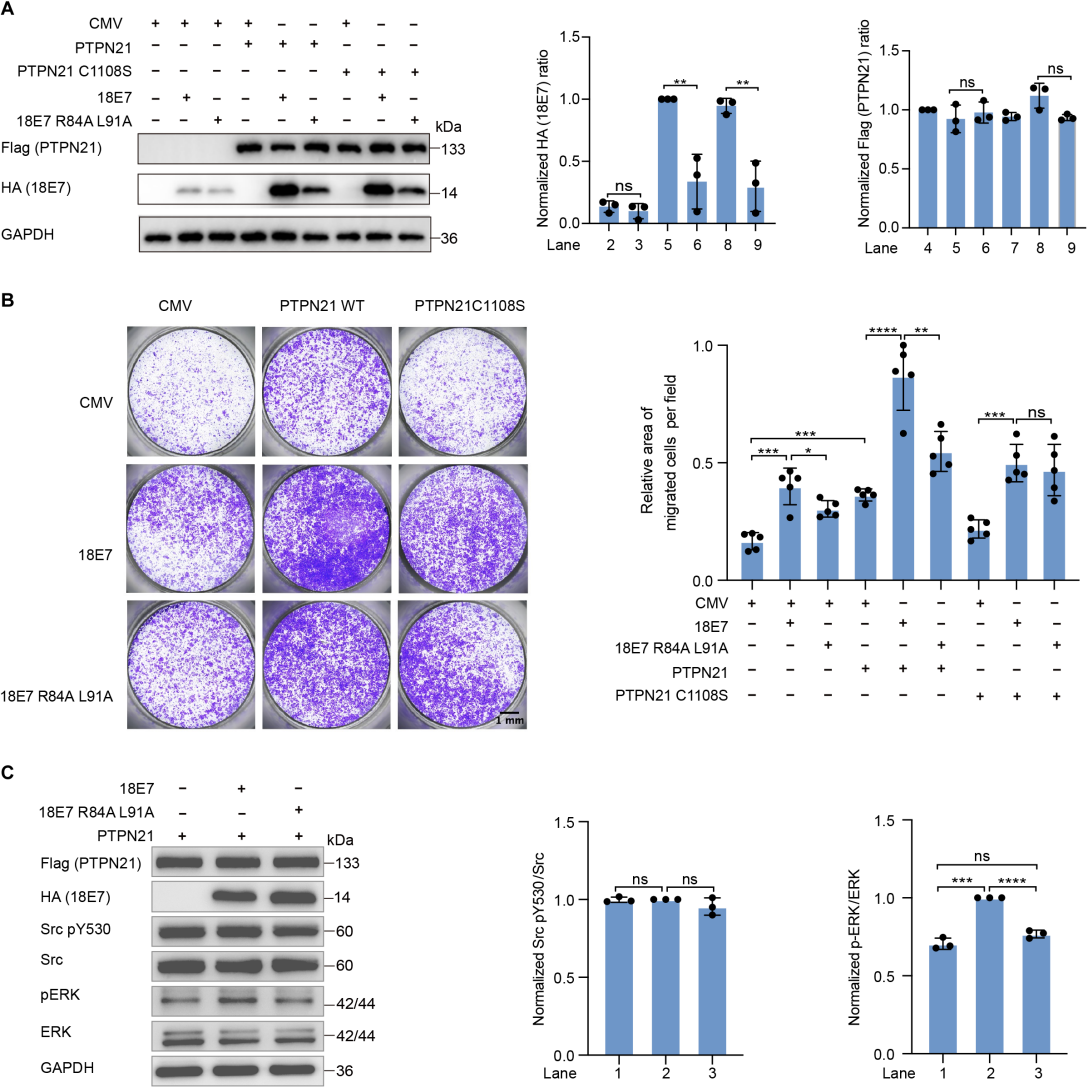
18E7 interacts with PTPN21 to promote cell migration. (**A**) PTPN21 up-regulates the protein level of 18E7. HEK293T cells were transiently transfected with empty vector (CMV), HA-18E7, or HA-18E7 R84A L91A plasmids (0.1 μg/μl) in combination with CMV, N-terminal Flag-tagged PTPN21, or PTPN21 C1108S plasmids (0.4 μg/μl) for 48 hours. For quantitative analysis, each lane of Western blots was sequentially shown as lanes 1 to lane 9 from left to right. Error bars represent ± SEM of *n* = 3 independent experiments. The relative HA values were calculated by normalizing to PTPN21 WT and 18E7. The relative Flag values were calculated by normalizing to PTPN21 WT. Statistical analysis was performed using Student’s *t* test (**P < 0.01 and ns *P* > 0.05). (**B**) Transwell assays were used to assess the migration abilities of HEK293T cells transiently transfected with CMV, HA-18E7, or 18E7 R84A L91A plasmids (0.1 μg/μl) in combination with CMV, N-terminal Flag-tagged PTPN21, or PTPN21C1108S plasmids (0.4 μg/μl) for 48 hours, related to (A). Cell colonies were visualized by Crystal Violet staining. Data assessed by Student’s *t* test. ****P < 0.0001, ***P < 0.001, **P < 0.01, *P < 0.05, ns *P* > 0.05. Scale bar, 1 mm. Magnification, ×4. (**C**) WT HPV18 E7 but not the R84A L91A resulted in more ERK activation in the presence of PTPN21. HEK293T cells were transiently transfected with N-terminal Flag-tagged PTPN21 (0.4 μg/μl) and 18 E7 (0.1 μg/μl) or 18 E7 R84A L91A mutant plasmids (1 μg/μl) for 48 hours. The relative values were calculated by normalizing to total protein from three independent experiments. Statistical analysis was performed using Student’s *t* test (****P < 0.0001, ***P < 0.001, ns *P* > 0.05).

In HEK293T cells, cotransfection of PTPN21 with WT 18E7 increased the protein level of WT 18E7 several times higher than 18E7 R84A L91A mutant (Fig. 6A). In Transwell assays, HEK293T cell migration was increased by overexpression of 18E7, 18E7 R84A L91A, PTPN21, and PTPN21 C1108S (Fig. 6B). Cotransfection of WT PTPN21 and WT 18E7 resulted in the highest proportion of cell migration, indicating that PTPN21 and 18E7 synergistically promoted cell migration (Fig. 6B). To further analyze the synergistic effect of PTPN21 and 18E7 on ERK, after balancing the protein level of 18E7 and 18E7 R84A L91A, cotransfection of PTPN21 with WT 18E7 led to more ERK phosphorylation than E7 R84A L91A mutant (Fig. 6C), which is in agreement with the reported protumor activity of PTPN21 in HPV18 E7 expressing Hela and HaCaT cells (*63*). On the other hand, in CCK-8 assays overexpression of 18E7 and PTPN21 in 293T cells did not significantly affect cell proliferation (fig. S7A).

## DISCUSSION

PTPN21 is a relatively large protein with two structured domains (FERM and PTP) and a long intrinsically disordered region in the middle. It is less well-studied compared to other tyrosine phosphatases like PTP1B or the closely related PTPN14. PTPN21 is known to be up-regulated in several types of cancer cells, and its mutations are found in many patients with cancer (*6*, *13*, *16*–*19*, *67*, *68*). In addition, because of the presence of E1067, conflicting results were reported by different groups regarding its phosphatase activity. To explore the working mechanism of PTPN21, we carried out structural and biochemical studies of the PTPN21 protein.

PTPN21 FERM domain was reported to be important for PTPN21 membrane localization and mediate interactions with Src kinase, EGFR, and KIF1C to regulate cell adhesion, scattering, and migration (*3*, *5*, *7*–*9*). Our results demonstrate that the FERM domain is able to associate with certain phosphatidylinositol phosphates through a patch of positively charged residues between its F1 and F3 lobes (Fig. 1, E to K, and fig. S2H). Fortuitously, two MPD molecules were buried in the hydrophobic pockets of the F2 lobe. MPD binding caused the PTPN21 FERM Y158 region to adopt a different conformation. Because of this altered conformation, we observed two kinds of FERM-FERM contacts involving Y158 (fig. S2, C to G). Although the purified FERM domain exists as a monomer, we could not rule out the possibility that FERM domains may self-associate after binding certain factors through the MPD-accommodating pockets.

PTPN21’s phosphatase activity is required in vivo as demonstrated by several studies (*3*, *5*, *6*, *18*, *69*). Our in vitro analysis clearly showed that PTPN21 PTP is active toward phosphorylated tyrosine substrates; however, its activity is indeed very weak compared to other phosphatases with normal WPD loops (*37*). In HEK293T cells, overexpression of PTPN21 resulted in a small reduction of Src pY530 phosphorylation; however, the difference between WT PTPN21 and C1108S was not significant (fig. S3C and Fig. 4N), and it is possible that PTPN21’s activity is under tight regulation and is only active under specific conditions.

Through biochemical assays, we found that PTPN21 FERM interacts with PTP and negatively regulates its phosphatase activity in vitro and in cells. The FERM-PTP complex structure allowed us to identify critical interface residues, and mutation of these residues led to increased ERK activation (Fig. 4, C to F and N, and fig. S5, E and F). These mutations only activated ERK in the context of WT PTP but not the catalytic inactive C1108S or R1114Q mutant, indicating that PTPN21’s phosphatase activity is indispensable (Fig. 4N and fig. S5E). Together, the weak intrinsic phosphatase activity and the autoinhibition by FERM may partially explain the few reported PTPN21 substrates in literature.

The overall structure of the phosphatase domain of PTPN21 is similar to classic tyrosine phosphatases like PTP1B. The CH/π switch mechanism discovered in PTP1B (*30*, *70*), which is critical for positioning the WPD loop, also applies to PTPN21 as all the corresponding residues are the same in both proteins. PTP1B has an α7 helix that is responsible for allosterically controlling PTP1B phosphatase activity, and deletion of α7 resulted in a 40% loss of PTP1B activity (*70*, *71*). The flexible α7 is believed to act by interacting with α3 and α6 to stabilize the WPD loop in a closed conformation, and the intersection of helices α3, α6, and α7 formed the allosteric binding pocket (fig. S8A) (*70*, *71*). When an inhibitor like benzofuran binds to this pocket, α7 is disordered and the WPD loop is trapped in the open conformation (fig. S8B) (*52*). Unlike PTP1B, PTPN21 lacks α7, which may to some extent contribute to its lower activity. Furthermore, the FERM domain interacts with α3, and it may act like allosteric inhibitors to trap the WPD loop in the open conformation and inhibits PTP phosphatase activity (fig. S8B).

HPV18 E7 protein binds to the same location as FERM but with 2000-fold higher affinity. As expected, the tight binding of 18E7 prevented FERM binding to PTP (Fig. 5, F and G). In HEK293T cells, we found that overexpression of PTPN21 led much higher protein level of 18E7 protein than a 18E7 mutant defective in binding PTPN21 (Fig. 6A). After adjusting the protein expression levels, WT 18E7 resulted in more ERK phosphorylation than the mutant defective in binding PTP (Fig. 6C). This is likely due to 18E7’s ability to compete with FERM and force PTPN21 to stay in the open conformation. In Transwell assays, cotransfection of PTPN21 WT and WT 18E7 resulted in the most cell migration, indicating that PTPN21 and 18E7 synergistically promoted cell migration (Fig. 6B), which is consistent with previous studies (*63*). Together, our results revealed that additional roles viral oncoprotein 18E7 might play in tumorigenesis.

In vitro, CR3^18E7^ binding or the presence of FERM (mini PTPN21) leads to weaker phosphatase activity (Fig. 5I); however, in 293T cells, 18E7 overexpression results in more ERK activation (Fig. 6C), thus it is possible that FERM might have additional roles in suppressing PTPN21 activity in cells, keeping FERM away (open conformation) by 18E7 overcomes the small reduction in PTP activity caused by 18E7. This is consistent with the results obtained from FERM-PTP interface mutations.

**Fig. 7.**
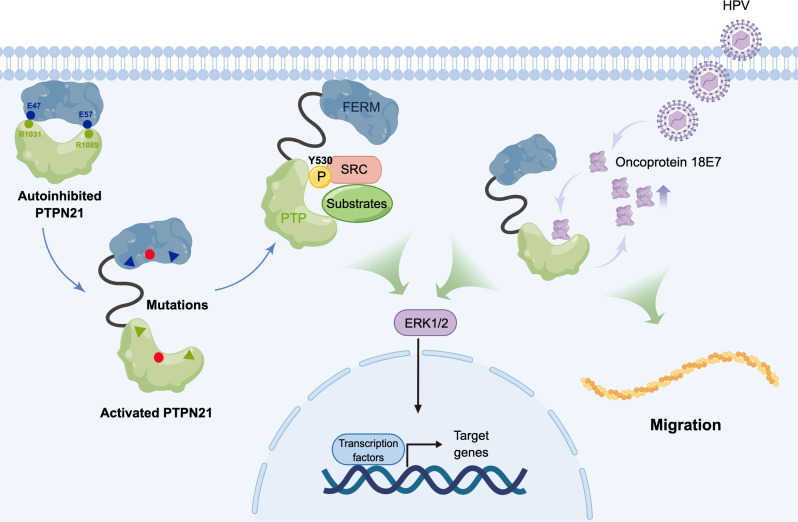
A working model of PTPN21 in oncogenic ERK activation. The FERM domain of PTPN21 associates with membrane lipids. Normally, PTPN21 is in an auto-inhibited state, where the FERM domain interacts with the PTP domain and stabilizes the WPE loop in the open (inactive) conformation. Mutations in the FERM-PTP interface including cancer-associated mutations disrupt FERM-PTP binding and free the PTP domain, leading to downstream ERK activation. In addition, when HPV infects cells, oncoprotein E7 binds to PTP at sites similar to FERM with high affinity and prevents FERM from binding to PTP, leaving PTPN21 in an open state to promote ERK signaling. E7 Binding to PTPN21 also leads to elevated protein level of E7, thereby synergistically promoting cell migration with PTPN21.

In conclusion, our study demonstrates that PTPN21 is autoinhibited by FERM-PTP interaction, and mutations or viral proteins disrupting the interaction between FERM and PTP would lead to increased PTPN21 activity and downstream ERK activation (Fig. 7). Our current work focusing on the FERM and PTP domains of PTPN21 provides a molecular framework for understanding the role of PTPN21 in various cellular processes. These findings are likely extendable to other FERM-containing tyrosine phosphatases like PTPN14 and PTPN3, which opens up avenues for further mechanistic studies.

While PTPN21 FERM mutations (E47A and E57A) and 18E7 binding both lead to the open conformation of PTPN21, coimmunoprecipitation between PTPN21 and endogenous Src in HEK293T cells showed that PTPN21 with E47A E57A mutation pulled down more Src than PTPN21 WT (fig. S9A); in contrast, 18E7 showed no significant effect on PTPN21-Src interaction (fig. S9B). It is possible that PTPN21 in the open conformation associates better with Src, but the physical presence of E7 bound to the PTP domain mitigates the enhanced association effect. The detailed interaction mechanism between PTPN21, Src, and E7 warrants further investigation. In addition, the middle region between the two PTPN21 domains is predicted to be largely disordered and is more difficult to study. Some of the residues in this disordered region have been demonstrated to interact with WW domains (*72*), and clinical studies have reported leukemia patients with quite a few mutations in this region (*17*); future studies will be required to elucidate how the middle region works together with the FERM and PTP domains to carry out its function.

## MATERIALS AND METHODS

### Materials and antibodies

High-glucose Dulbecco’s modified Eagle’s medium (HG-DMEM), fetal bovine serum (FBS), penicillin, and streptomycin were ordered from Gibco. The EnzChek Phosphatase Assay Kit (catalog no. E12020) and DiFMUP (catalog no. D6567) were purchased from Thermo Fisher Scientific Inc. The Malachite Green Phosphate Detection Kit (catalog no. DY996) was obtained from R&D Systems. Peptide Src pY530 (sequence: STEPQpY QPGENL) was synthesized by ChinaPeptides Co. Ltd.

The following antibodies were used for Western blot experiments: rabbit anti-p44/42 mitogen-activated protein kinase (MAPK) (ERK1/2; Cell Signaling Technology, catalog no. 4695), rabbit anti-phosphothreonine (202)/tyrosine (204)-p44/42 MAPK (pERK1/2; Cell Signaling Technology, catalog no. 4370), rabbit anti-Src (Cell Signaling Technology, catalog no. 2109), rabbit anti–hemagglutinin (HA) (Cell Signaling Technology, catalog no. 3724), rabbit anti-Flag (Cell Signaling Technology, catalog no. 14793), rabbit anti-Src (phospho Y529/530) (Abcam, catalog no. ab32078), rabbit anti–glyceraldehyde phosphate dehydrogenase (Beyotime Biotechnology, catalog no. AF5009), and goat anti-rabbit IgG/horseradish peroxidase (HRP) (Beyotime Biotechnology, catalog no. A0208).

### Recombinant protein expression and purification

The DNA sequence encoding the FERM domain (residues 21 to 319) or the PTP domain (residues 872 to 1174) was cloned into a modified pRSFDuet-1 vector with an N-terminal His-sumo tag (Novagen). The plasmids were transformed into *Escherichia coli* Rosetta (DE3) for overexpression. Protein expression was induced by adding 0.2 mM isopropyl-β-d-thiogalactopyranoside to LB cultures at optical density (OD) at 0.6, and the cells were further cultured for 16 to 18 hours at 18°C. The cells were collected and disrupted by French press in a lysis buffer [20 mM tris-HCl (pH 8.0), 300 mM NaCl, 10% glycerol, 0.3 mM Tris(2-carboxyethyl)phosphine (TCEP), and 0.1 mM phenylmethylsulfonyl fluoride (PMSF)]. The supernatant was loaded onto a Nickel-Nitrilotriacetic acid (Ni-NTA) resin gravity column and washed three times with lysis buffer. The recombinant protein was eluted with the elution buffer [20 mM tris-HCl (pH 8.0), 300 mM NaCl, 250 mM imidazole, 10% glycerol, 0.3 mM TCEP, and 1 mM PMSF]. His-Ulp1 was added to the eluted protein sample to remove the His-sumo tag, and the mixture was dialyzed against the lysis buffer to remove excess imidazole. The resulting sample was then applied to a Ni-NTA resin column, and the flow-through was collected. The flow-through sample was loaded into a source Q column equilibrated with source Q buffer A [20 mM tris-HCl (pH 8.0), 20 mM NaCl, 10% glycerol, and 0.3 mM TCEP], and the bound proteins were eluted with source Q buffer B [20 mM tris-HCl (pH 8.0), 1 M NaCl, 10% glycerol, and 0.3 mM TCEP]. The eluted protein fractions were further purified by gel filtration chromatography using a Superdex 200 increase column (Cytiva) equilibrated with gel filtration buffer [20 mM tris-HCl (pH 8.0), 200 mM NaCl, and 0.3 mM TCEP]. Other proteins (CR3^18E7^, mini PTPN21), different mutants, and proteins with enhanced green fluorescent protein (EGFP) tag used in MST analysis were expressed and purified similarly.

For GST fusion protein expression, DNA sequences of the FERM or PTP domains were cloned into a pGEX-6P-1 vector with an N-terminal GST tag and expressed in *E. coli* Rosetta (DE3). They were purified by glutathione-Sepharose beads and further purified by gel filtration chromatography using a Superdex 200 increase column (Cytiva) in a buffer containing 20 mM tris-HCl (pH 8.0), 200 mM NaCl, and 0.3 mM TCEP.

Flag-tagged Src or the Src^ΔSH2^ mutant was cloned into a pcDNA3.1 vector with an N-terminal Flag tag and expressed in HEK293T cells by transient transfection. Cells were collected and lysed with lysis buffer [20 mM tris-HCl (pH 7.5), 150 mM NaCl, 10% glycerol, 0.5% Triton X-100, and 0.5 mM EDTA supplemented with protease inhibitors (Topscience, catalog no. C0001), phosphatase inhibitors (Topscience, catalog no. C0004), and UltraNuclease (Yeasen Biotechnology, catalog no. 20156ES)]. The lysate was cleared by centrifugation at 10,000*g* for 15 min. The supernatant was incubated with anti-Flag M2 agarose beads (Sigma-Aldrich) for 1 hour. Following 3× column volumes of wash with a washing buffer [20 mM tris-HCl (pH 7.5), 150 mM NaCl, 10% glycerol, 0.5 mM EDTA, and 0.3 mM TCEP], Flag-tagged protein was eluted with the elution buffer [20 mM tris-HCl (pH 7.5), 150 mM NaCl, 10% glycerol, 0.5 mM EDTA, 0.3 mM TCEP, and 35 μM 3× Flag peptide]. Eluted protein was concentrated and subjected to in vitro phosphatase assay.

### Crystallization

Crystal screenings were performed by hanging-drop vapor-diffusion methods at 4°C. Crystals of the FERM domain were grown by mixing 1.2 μl of FERM (15 mg/ml) with 1.2 μl of reservoir solution [0.1 M sodium acetate (pH 5.0) and 25% MPD]. Crystals of the PTP C1108S domain were obtained by mixing 1.2 μl of PTP (15 mg/ml) with 1.2 μl of reservoir solution containing 0.1 M Hepes (pH 7.5), 250 mM NaI, 19% (w/v) Polyethylene Glycol 3350 (PEG3350), and 0.1 mM TCEP. To obtain crystals of PTP C1108S in complex with Src pY530 peptide, twofold molar excess of Src pY530 peptide was added to PTP C1108S (18 mg/ml), and 1.2 μl of the mixture was combined with 1.2 μl of reservoir solution [0.08 M Hepes (pH 7.5), 250 mM NaI, 50 mM NaCl, 17% (w/v) PEG3350, and 0.1 mM TCEP]. Crystals of mini PTPN21 were obtained by mixing 1.2 μl of mini PTPN21 (15 mg/ml) with 1.2 μl of reservoir solution consisting of 0.08 M Hepes (pH 7.5), 50 mM NaCl, 10% (w/v) PEGMME2000, 0.01 M spermidine, and 0.1 mM TCEP. To collect data at a low temperature, crystals were soaked briefly in a cryoprotecting solution containing all of the components of the reservoir solution supplemented with 20 to 25% glycerol or ethylene glycol and flash-frozen in liquid nitrogen for x-ray diffraction.

### Structure determination and refinement

Diffraction data were collected at beamlines BL19U1 and BL17U1 of the Shanghai Synchrotron Radiation Facility. Data were indexed, integrated, and scaled using the XDS, CCP4 program Pointless, and Aimless (*73*–*75*). The structures of PTPN21 FERM and PTPN21 PTP C1108S were determined by molecular replacement with Phaser (*76*), using the structure of avian FERM domain (PDB: 2AL6) or PTPN14 PTP (PDB: 2BZL) as the starting model, respectively. The structural models were built using Coot (*77*) and refined using PHENIX (*78*). The structures of PTPN21 PTP-Src pY530 complex and mini PTPN21 were determined by molecular replacement using PTP as a search model. The linker residues in mini PTPN21 were not observed in the electron density map, indicating they are flexible and not affecting protein interaction. The statistics of the data collection and refinement are shown in table S1. Figures were generated using PyMOL (The PyMOL Molecular Graphics System, Version 2.0 Schrödinger, LLC), the PDBsum website (http://ebi.ac.uk/thornton-srv/databases/pdbsum/), and Figdraw (www.figdraw.com, for the graphical model).

### Cell culture and transfections

HEK293T cells were cultured in HG-DMEM supplemented with 10% FBS, penicillin (100 U/ml), and streptomycin (100 mg/ml) at 37°C and 5% CO_2_. HEK293T cells were transfected at approximately 70 to 75% confluency using PolyJet DNA (SignaGen Laboratories, catalog no. SL100688) according to the manufacturer’s instructions, and cells were treated and harvested for experimentation 48 hours posttransfection.

### In vivo phosphatase assays and immunoblotting

For the Flag epitope, *PTPN21* cDNA (WT and mutants) were subcloned into a pcDNA3.1 vector (Invitrogen) with an N-terminal Flag-tag. These plasmids were transformed into HEK293T cells for overexpression. Cells were washed twice with ice-cold PBS and lysed in 200 μl per 6-cm dish of ice-cold radioimmunoprecipitation assay lysis buffer (Solarbio, catalog no. R0020), supplemented with EDTA-free protease inhibitors, phosphatase inhibitors, and UltraNuclease by orbital shaking on ice. Total protein concentrations of cell lysates were quantified by BCA assay. Twenty-five to 50 μg of lysate was mixed with 5× sample loading buffer and incubated at 95°C for 5 min.

For immunoblotting, proteins were separated by 10% SDS–polyacrylamide gel electrophoresis (SDS-PAGE) and transferred to 0.2-μm reinforced nitrocellulose membranes (GE Healthcare) by wet transfer. The membranes were blocked in 5% (w/v) bovine serum albumin (BSA) in TBST [20 mM tris-HCl (pH 7.6), 137 mM NaCl, and 0.2% (v/v) Tween 20] for 1 hour at RT and then probed with the appropriate primary antibodies overnight at 4°C. After incubation, membranes were washed and incubated with the appropriate species-specific HRP-conjugated anti-IgG secondary antibody for 1 hour at room temperature (RT). Proteins were detected using a chemiluminescence imaging system (Clinx Science).

### In vitro phosphatase assay

For DiFMUP phosphatase assay, PTP^PTPN21^ or mutants were incubated with the substrate DiFMUP in a 100-μl reaction buffer containing 50 mM Hepes (pH 7.0), 150 mM NaCl, 1 mM EDTA, 2 mM dithiothreitol (DTT), 2% glycerol, and 1.5 mM BSA in a 96-well flat-bottom plate at RT. Fluorescence from the excitation of hydrolyzed DiFMUP and the standard DiFMU was detected in a fluorescence microplate reader (Varioskan Flash, Thermo Fisher Scientific) using 358-nm excitation and 455-nm emission wavelengths. For determination of the appropriate enzyme concentration, different concentrations of PTP^PTPN21^ ranging from 0.6 to 6 nM were incubated with 40 μM DiFMUP. For kinetic analysis, PTP^PTPN21^ (5 nM) was incubated with varying concentrations of DiFMUP ranging from 5 to 400 μM in buffers with different pH {150 mM NaCl, 1 mM EDTA, 2 mM DTT, 2% glycerol, 1.5 mM BSA, and 50 mM pH buffers [MES (pH 6.0), Hepes (pH 7.0), or tris-HCl (pH 8.0)]}. Besides, a fixed concentration of enzyme (5 nM), except PTP Q1156A (25 nM) and PTP Q1152A (500 nM), was added to varying concentrations of DiFMUP ranging from 5 to 400 μM at pH 7.0. To test the effect of inositol phosphate on enzymatic activity, 1 μM ins(1,3)P_2_ or ins(1,5)P_2_ was incubated with miniPTPN21 or miniPTPN21 E47A E57A (5 nM) for 10 min and then added to varying concentrations of DiFMUP ranging from 5 to 300 μM at pH 7.0. In addition, to test the effect of inositol phosphate on enzymatic activity with CR3^18E7^,18E7 or mutants, 1 μM ins(1,3)P_2_ or ins(1,5)P_2_ was incubated with miniPTPN21 (5 nM) and then added to 5 nM 18E7, CR3^18E7^ or mutants, followed by added to varying concentrations of DiFMUP ranging from 5 to 300 μM at pH 7.0. Raw data were fitted using linear regression in GraphPad Prism software (v9.0) to determine the enzyme concentration range linear with velocity. Initial velocities were processed with nonlinear regression, and kinetic constants (*V*_max_ and *K*_m_) were calculated using the Michaelis-Menten equation in GraphPad Prism. *K*_cat_ values were calculated using the equation *K*_cat_ = *V*_max_/[E_T_].

For *p*NPP phosphatase assay, PTP^PTPN21^ (17.7 μM) was added with 46.8 mM *p*NPP substrate (Meilunbio, catalog no. MB3188) in a 100-μl reaction buffer with different pH {150 mM NaCl, 1 mM EDTA, 2 mM DTT, 2% glycerol, 1.5 mM BSA, and 50 mM pH buffers [MES (pH 6.0), Hepes (pH 7.0), or tris-HCl (pH 8.0)]} in a 96-well flat-bottom plate at RT for 20 min. Product formation was monitored by measuring absorption at 405 nm in a fluorescence microplate reader (Varioskan Flash, Thermo Fisher Scientific). The results from three repeated experiments were analyzed using GraphPad Prism software (v9.0).

For Src pY530 peptide phosphatase activity assay, 2.68 μM WT PTP or miniPTPN21 alone or in the presence of an equal molar amount of CR3^18E7^ or CR3^18E7^ R84A L91A was mixed with 268 μM Src pY530 peptide in a 250-μl reaction [50 mM Hepes (pH 7.5), 75 mM NaCl, and 1 mM DTT] and incubated at 30°C for 15 min. The release of phosphate was measured by absorbance at 360 nm using the Malachite Green Phosphate Detection Kit following the manufacturer’s protocol. For phosphatase activity analysis against recombinant Src, 8 nM WT PTP or mutants were incubated with 0.8 μM recombinant Flag-tagged Src or the Src^ΔSH2^ mutant in a 50-μl reaction buffer [50 mM Hepes (pH 7.0), 80 mM NaCl, 100 μM EDTA, 1 mM DTT, 2 mM MgCl_2,_ 1.5 mM BSA, and 2% glycerol] and incubated at 30°C for 1 hour, respectively. The reaction was stopped by adding 1× SDS-PAGE sample buffer and analyzed by immunoblotting using specific antibodies.

### PIP-strips assay

Membrane lipid binding analysis of Flag-tagged FERM or mutant was conducted using PIP-StripsTM membranes (Thermo Fisher Scientific), with each nitrocellulose membrane containing 100 pmol of 15 different phospholipids and a blank sample. PIP strip was blocked in TBST with 3% fatty acid–free BSA for 1 hour at RT, followed by adding Flag-tagged FERM (5 μg/ml) or mutant at 4°C overnight. The membrane was then washed three times with TBST and blotted with an anti-Flag rabbit monoclonal antibody (1:1000; Cell Signaling Technology, catalog no. 14793S) overnight at 4°C. After washing again three times with TBST, the membrane was then incubated with the appropriate species-specific HRP-conjugated anti-IgG secondary antibody (1:5000; Beyotime Biotechnology, catalog no. A0208) for 1 hour at RT. Proteins were detected using a chemiluminescence imaging system (Clinx Science).

### Inactivation of PTP variants by H_2_O_2_

To measure the susceptibility of PTPN21 PTP variants to oxidation, PTP WT, PTP C1155S, and PTP C1070S (5 nM) were incubated with different concentrations of DiFMUP varying from 5 to 300 μM in reaction buffers with increasing concentrations of H_2_O_2_ (0, 50, 100, and 400 μM). The fluorescence signal of DiFMUP was measured in a fluorescence microplate reader (Varioskan Flash, Thermo Fisher Scientific).

### GST pulldown

GST-tagged FERM domain (60 μM; residues 21 to 319) or GST-FERM E47A E57A mutant was incubated with an equal molar amount of PTP^PTPN21^ (residues 872 to1174) in a 40-μl binding reaction containing 20 mM tris-HCl (pH 8.0), 75 mM NaCl, 0.3 mM TCEP, and 2% glycerol. After 30-min incubation at 4°C, 15-μl glutathione-agarose beads were added to each reaction followed by 1-hour incubation at 4°C with rotation. Beads were then washed three times with binding buffer, and 50 μl of 1× SDS loading buffer was added to each reaction. Beads with bound protein were boiled at 95°C for 10 min and analyzed by 12% SDS-PAGE. GST was used as a control for nonspecific binding to GST. Other GST pulldown experiments were carried out similarly.

### MST titration

EGFP tagged to the C terminus of PTPN21 PTP, FERM, mutants, or miniPTPN21 contribute to the thermophoresis signal upon targets. The protein target was fixed at 80 nM. The targets and ligands were diluted with a reaction buffer containing 20 mM Hepes (pH 7.0), 150 mM NaCl, 0.3 mM TCEP, and 1.5 mM BSA and loaded into MST NT.115 standard glass capillaries (NanoTemper Technologies). For WT PTP-EGFP with FERM binding experiments, FERM was titrated with different concentrations from 300 μM to 0.09 nM; for FERM-EGFP with Ins(1,3)P_2_ and Ins(1,5)P_2_ binding experiments, Ins(1,3)P_2_ or Ins(1,5)P_2_ was respectively titrated with different concentrations from 500 μM to 0.15 nM or from 150 μM to 0.045 nM; for the effect of inositol phosphate on FERM-EGFP and PTP binding, 1 μM ins(1,3)P_2_ or ins(1,5)P_2_ was incubated with FERM-EGFP for 10 min, and PTP was titrated with different concentrations from 150 μM to 0.045 nM. For CR3^18E7^with PTP-EGFP or mini PTPN21-EGFP binding experiments, CR3^18E7^ was respectively titrated with different concentrations from 1 μM to 0.0003 nM or from 2 μM to 0.0006 nM; for the effect of inositol phosphate on mini PTPN21-EGFP and 18E7 binding, 1 μM ins(1,3)P_2_ or ins(1,5)P_2_ was incubated with mini PTPN21-EGFP for 10 min, and 18E7 binding was titrated with different concentrations from 125 μM to 0.038 nM. After 10 min of incubation at RT, the samples were loaded into MST NT.115 standard glass capillaries. Data were analyzed and fit to the *K*_D_ model using MO. Affinity software (version 2.3) (NanoTemper Technologies) was provided by the manufacturer. Other MST experiments were carried out similarly.

### Nano-DSF measurement

A tryptophan-fluorescence-based thermal unfolding experiment was performed using Prometheus NT.48 nano-DSF (Nanotemper Technologies). The capillaries containing 10 μl of protein (10 μM) were loaded into the machine. Samples were then heated at a rate of 1°C/min from 20° to 95°C, and protein unfolding at each temperature monitored by measurement of fluorescence intensities at emission wavelengths of 330 and 350 nm was measured. The ratio of fluorescence intensities at 350 and 330 nm was used as a function of temperature to determine the protein inflection point, which is the temperature at which half of the protein is unfolded.

### Cell proliferation assay

HEK293T cells were transiently transfected with plasmids coding PTPN21 and/or 18E7. After expressing for 48 hours, cells were collected and seeded evenly in a 48-well plate, at 10,000 cells per well, and cultured at 37°C and 5% CO_2_ for 48 hours. Ten microliters of CCK-8 reagent was added to each well, and the OD_450_ of each well was detected by a BioTek Synergy HT plate reader (BioTek, USA).

### Migration assay

Transwell chambers with 8.0-μm pore polycarbonate membrane (Corning, catalog no. CLS3422) were used to analyze cell migration. HEK293T cells transiently expressing PTPN21 and/or 18E7 were collected and suspended in DMEM medium supplemented with 1% FBS, 200 μl of medium containing 2 × 10^4^ cells were added to the upper chamber, and 500 μl DMEM supplemented with 20% FBS was subsequently added to the wells of the plates. After incubation at 37°C and 5% CO_2_ for 36 hours, migrated cells was fixed with 4% paraformaldehyde and then stained with 1% crystal violet. Cell colonies were imaged using a Leica SC1200C microscope and quantified using ImageJ software.

### Coimmunoprecipitation assay

Flag-tagged PTPN21 (1 μg/μl; WT or mutant) plasmids alone or in the presence of HA-tagged 18E7 plasmids (0.25 μg/μl) were tranfected into HEK293T cells. Forty-eight hours after transfection, cells were washed twice with ice-cold PBS and lysed in 250 μl of ice-cold lysis buffer [20 mM tris-HCl (pH 8.0), 0.15 M NaCl, 10% glycerol, and 0.5% NP-40], supplemented with EDTA-free protease inhibitors (Topscience, catalog no. C0001), phosphatase inhibitors (Topscience, catalog no. C0004), and UltraNuclease (Yeasen Biotechnology, catalog no. 20156ES). Cell lysates were centrifuged at 700*g* for 5 min, and the supernatants were incubated with Flag beads at 4°C for 4 hours. The beads were washed three times with lysis buffer, mixed with 1× sample loading buffer, and boiled at 95°C for 5 min for Western blotting.
