# Supplementary material for: Structural analysis of PTPN21 reveals a dominant-negative effect of the FERM domain on its phosphatase activity
Source: Sci Adv. 2024 Feb 28;10(9):eadi7404. doi: 10.1126/sciadv.adi7404 (PMC10901363; doi:10.1126/sciadv.adi7404)
Supplement: Supplementary file 1 — Figs. S1 to S9 Table S1 [file sciadv.adi7404_sm.pdf]

Science Advances
AAAS

Supplementary Materials for

Structural analysis of PTPN21 reveals a dominant-negative effect of the FERM domain on its phosphatase activity

Lu Chen *et al.*

Corresponding author: Haowen Xiao, haowenxiaoxiao@zju.edu.cn; Chun Zhou, chunzhou@zju.edu.cn

Sci. Adv. **10**, eadi7404 (2024)
DOI: 10.1126/sciadv.adi7404

This PDF file includes:

Figs. S1 to S9
Table S1

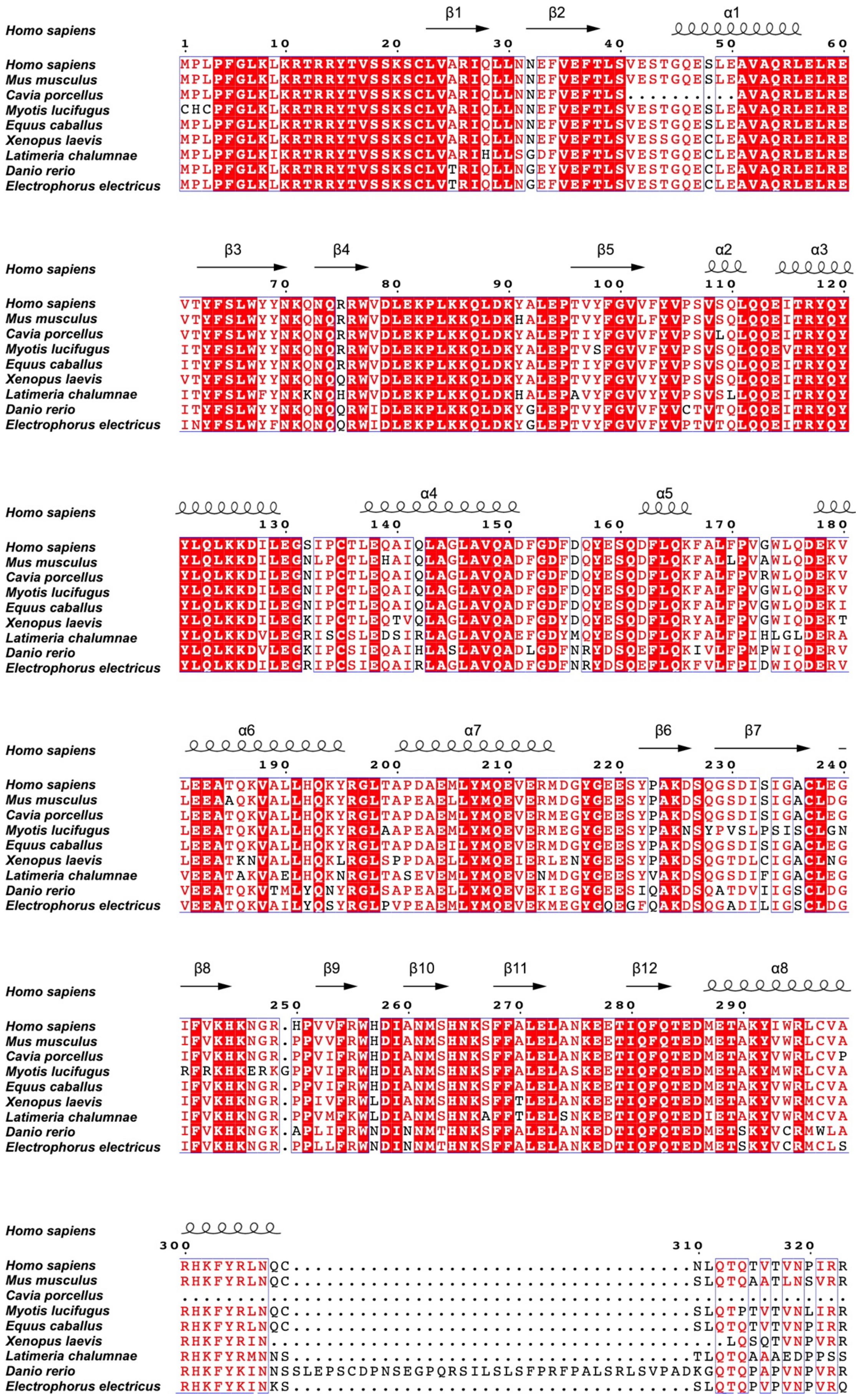
β1 β2 α1
β3 β4 β5 α2 α3
α4 α5
α6 α7 β6 β7
β8 β9 β10 β11 β12 α8
Homo sapiens
Mus musculus
Cavia porcellus
Myotis lucifugus
Equus caballus
Xenopus laevis
Latimeria chalumnae
Danio rerio
Electrophorus electricus

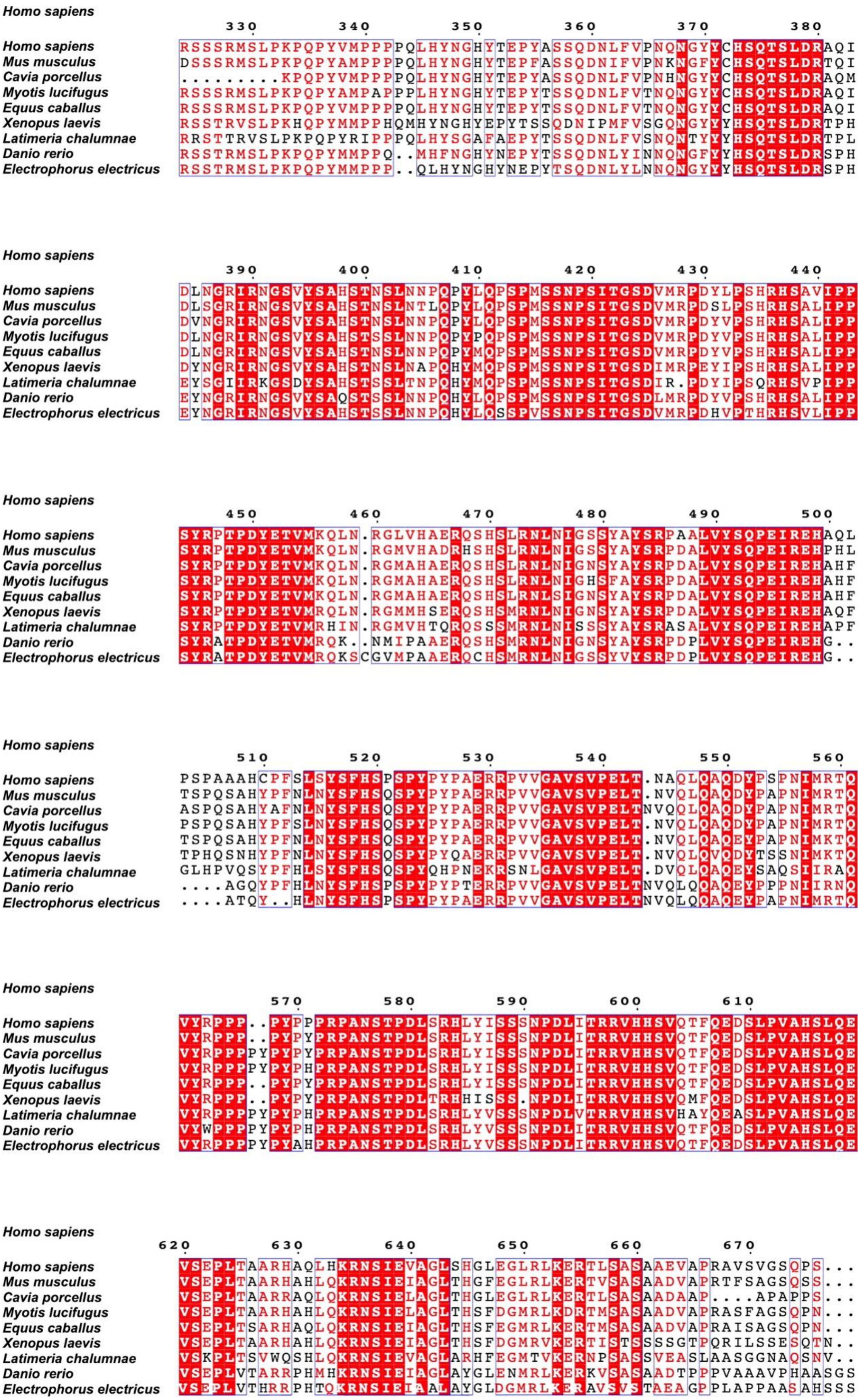
Homo sapiens
330 340 350 360 370 380
Homo sapiens RSSSRMSLPKPQPYVMPPPPQLHYNGHYTEPYASSQDNLFVPNQNGYYCHSQTSLDRAQI
Mus musculus DSSSRMSLPKPQPYAMPPPPQLHYNGHYTEPFASSQDNIFVPNKNGFYCHSQTSLDRTQI
Cavia porcellus .........KPQPYVMPPPPQLHYNGHYTEPYASSQDNLFVPNHNGYYCHSQTSLDRAQM
Myotis lucifugus RSSSRMSLPKPQPYAMPAPPPLHYNGHYTEPYTSSQDNLFVTNQNGYYCHSQTSLDRAQI
Equus caballus RSSSRMSLPKPQPYVMPPPPQLHYNGHYTEPYTSSQDNLFVTNQNGYYCHSQTSLDRAQI
Xenopus laevis RSSTRVSLPKHQPYMMPPHQMHYNGHYEPYTSSQDNIPMFVSGQNGYYYHSQTSLDRTPH
Latimeria chalumnae RRSTTRVSLPKPQPYRIPPPQLHYSGAFAEPYTSSQDNLFVSNQNTYYYHSQTSLDRTPL
Danio rerio RSSTRMSLPKPQPYMMPPQ..MHFNGHYNEPYTSSQDNLYINNQNGFYYHSQTSLDRSPH
Electrophorus electricus RSSTRMSLPKPQPYMMPPP..QLHYNGHYNEPYTSQDNLYLNNQNGYYYHSQTSLDRSPH
Homo sapiens
390 400 410 420 430 440
Homo sapiens DLNGRIRNGSVYSAHSTNSLNNPQPYLQPSPMSSNPSITGSDVMRPDYLPSHRHSAVIPP
Mus musculus DLSGRIRNGSVYSAHSTNSLNTLQPYLQPSPMSSNPSITGSDVMRPDSLPSHRHSALIPP
Cavia porcellus DVNGRIRNGSVYSAHSTNSLNNPQPYLQPSPMSSNPSITGSDVMRPDYVPSHRHSALIPP
Myotis lucifugus DLNGRIRNGSVYSAHSTNSLNNPQPYPQPSPMSSNPSITGSDVMRPDYVPSHRHSALIPP
Equus caballus DLNGRIRNGSVYSAHSTNSLNNPQPYMQPSPMSSNPSITGSDVMRPDYVPSHRHSALIPP
Xenopus laevis DYNGRIRNGSVYSAHSTNSLNAPQHYMQPSPMSSNPSITGSDIMRPEYIPSHRHSALIPP
Latimeria chalumnae EYSGIIRKGSDYSAHSTSSLTNPQHYMQPSPMSSNPSITGSDIR.PDYIPSQRHSVPIPP
Danio rerio EYNGRIRNGSVYSAQSTSSLNNPQHYLQPSPMSSNPSITGSDLMRPDYVPSHRHSALIPP
Electrophorus electricus EYNGRIRNGSVYSAHSTSSLNNPQHYLQSSPVSSNPSITGSDVMRPDHVPTHRHSVLIPP
Homo sapiens
450 460 470 480 490 500
Homo sapiens SYRPTPDYETVMKQLN.RGLVHAERQSHSLRNLNIGSSYAYSRPAALVYSQPEIREHAQL
Mus musculus SYRPTPDYETVMKQLN.RGMVHADRHSHSLRNLNIGSSYAYSRPDALVYSQPEIREHPHL
Cavia porcellus SYRPTPDYETVMKQLN.RGMAHAERQSHSLRNLNIGNSYAYSRPDALVYSQPEIREHAHF
Myotis lucifugus SYRPTPDYETVMKQLN.RGMAHAERQSHSLRNLNIGHSFAYSRPDALVYSQPEIREHAHF
Equus caballus SYRPTPDYETVMKQLN.RGMAHAERQSHSLRNLSIGNSYAYSRPDALVYSQPEIREHAHF
Xenopus laevis SYRPTPDYETVMRQLN.RGMMHSERQSHSMRNLNIGNSYAYSRPDALVYSQPEIREHAQF
Latimeria chalumnae SYRPTPDYETVMRHIN.RGMVHTQRQSSSMRNLNISSSYAYSRASALVYSQPEIREHAPF
Danio rerio SYRATPDYETVMRQK..NMIPAAERQSHSMRNLNIGNSYAYSRPDPLVYSQPEIREHG..
Electrophorus electricus SYRATPDYETVMRQKSCGVMPAAERQCHSMRNLNIGSSYVYSRPDPLVYSQPEIREHG..
Homo sapiens
510 520 530 540 550 560
Homo sapiens PSPAAAHCPFSLSYSFHSPSPYPYPAERRPVVGAVSVPELT.NAQLQAQDYPSPNIMRTQ
Mus musculus TSPQSAHYPFNLNYSFHSQSPYPYPAERRPVVGAVSVPELT.NVQLQAQDYPAPNIMRTQ
Cavia porcellus ASPQSAHYAFNLNYSFHSQSPYPYPAERRPVVGAVSVPELTNVQLQAQDYPAPNIMRTQ
Myotis lucifugus PSPQSAHYPFSLNYSFHSQSPYPYPAERRPVVGAVSVPELT.NVQLQAQDYPAPNIMRTQ
Equus caballus TSPQSAHYPFNLNYSFHSQSPYPYPAERRPVVGAVSVPELT.NVQLQAQEYPAPNIMKTQ
Xenopus laevis TPHQSNHYPFNLNYSFHSQSPYPYQAERRPVVGAVSVPELT.NVQLQVQDYTSSNIMKTQ
Latimeria chalumnae GLHPVQSYPFHLSYSFHSQSPYQHPNEKRSNLGAVSVPELT.DVQLQAQEYSAQSIIRAQ
Danio rerio ....AGQYPFHLNYSFHSPSPYPYPTERRPVVGAVSVPELTNVQLQQAQEYPPPNIIRNQ
Electrophorus electricus ....ATQY..HLNYSFHSPSPYPYPAERRPVVGAVSVPELTNVQLQQAQEYPAPNIMRTQ
Homo sapiens
570 580 590 600 610
Homo sapiens VYRPPP..PYPPRPANSTPDLSRHLYISSSNPDLITRRVHHSVQTFQEDSLPVAHSLQE
Mus musculus VYRPPP..PYPYPRPANSTPDLSRHLYISSSNPDLITRRVHHSVQTFQEDSLPVAHSLQE
Cavia porcellus VYRPPPPYPYPYPRPANSTPDLSRHLYISSSNPDLITRRVHHSVQTFQEDSLPVAHSLQE
Myotis lucifugus VYRPPPPYPYPHPRPANSTPDLSRHLYISSSNPDLITRRVHHSVQTFQEDSLPVAHSLQE
Equus caballus VYRPPP..PYPYPRPANSTPDLSRHLYISSSNPDLITRRVHHSVQTFQEDSLPVAHSLQE
Xenopus laevis VYRPPP..PYPYPRPANSTPDLTRHHISSS.NPDLITRRVHHSVQMFQEDSLPVAHSLQE
Latimeria chalumnae VYRPPPPYPYPHPRPANSTPDLSRHLYVSSSNPDLVTRRVHHSVHAYQEASLPVAHSLQE
Danio rerio VYWPPPPYPYPHPRPANSTPDLSRHLYVSSSNPDLITRRVHHSVQTFQEDSLPVAHSLQE
Electrophorus electricus VYRPPPPYPYAHPRPANSTPDLSRHLYVSSSNPDLITRRVHHSVQTFQEDSLPVAHSLQE
Homo sapiens
620 630 640 650 660 670
Homo sapiens VSEPLTAARHAQLHKRNSIEVAGLSHGLEGLRLKERTLSASAAEVAPRAVSVGSQPS...
Mus musculus VSEPLTAARHAHLQKRNSIEIAGLTHGFEGLRLKERTVSASAADVAPRTFSAGSQSS...
Cavia porcellus VSEPLTAARRAQLQKRNSIELAGLTHGLEGLRLKERTLSASAADAAP....APAPPS...
Myotis lucifugus VSEPLTAARHAHLQKRNSIEIAGLTHSFDGMRLKDRTMSASAADVAPRASFAGSQPN...
Equus caballus VSEPLTSARHAQLQKRNSIEIAGLTHSFEGMRLKERTMSASAADVAPRAISAGSQPN...
Xenopus laevis VSEPLTAARHAHLQKRNSIEIAGLTHSFDGMRVKERTISTSSSSGTPQRILSSESQTN..
Latimeria chalumnae VSKPLTSVWQSHLQKRNSIEVAGLARHFEGMTVKERNPSASSVEASLAASGGNAQSNV..
Danio rerio VSEPLVTARRPHMHKRNSIEIAGLAYGLENMRLKERKVSASAADTPPPVAAAVPHAASGS
Electrophorus electricus VSEPLVTHRRPHTQKRNSIEIAALAYGLDGMRLKERAVSVSTAEAGPPLAPPAASAHSSS

```
Homo sapiens
                                  680       690       700       710       720
Homo sapiens             ...VFTERTQREGPEEAEGLRYGHKKSLSDATMLIHSSEEEEDEDFEEES....GARAPP
Mus musculus             ...VFSDKMKQEGTEEQEGGRYSHKKSLSDATMLIDSSEEDED.LEEDSS....REQAIS
Cavia porcellus          ...VCSNRAKQDGTEEQEGGRYGHKKSLSDATMLIHSSEEEEEEFEEDLK....VAAALP
Myotis lucifugus         ...VFPDRTRRGETAEQEGLRYGHSKSLSEATMLIHSSDEEEEPEEDSR....ARAVLS
Equus caballus           ...VFIERTKQEETDEQESLRYGHKKSLSDATMLIHSS.EEEEDFEEESK....AQAALP
Xenopus laevis           ...VIMERTKEEQGEDSERGGYGHKKSLSDATMLIHSSEDDDEEAEEDNR....TSITQA
Latimeria chalumnae      ...VFEHFKLQHGGSEPEEVRYGHKKSLSDATMLVHSSEEEEEEYMDDGSGAFVPRLPHP
Danio rerio              DINVVLEVSKPEDVGIKEDVFYGHKKSLSDATMLVHSSGEEEEFEDDSGR..........
Electrophorus electricus ELGVFLERTKAEETIIKEDVCYGHKKSLSDATMLVHSSGEEEEFEDDGGR..........

Homo sapiens
                         730       740       750       760       770       780
Homo sapiens             AR..AREPRPGLAQDPPG....CPRVLLAGPLHILEPKA...HVPDAEKRMMDSSPVRTT
Mus musculus             AVSEPRLTAAFSQELN...YPCASATPITGPLHIFEPKP...HVTEPEKRAKDISPVHLV
Cavia porcellus          TQPEPRLLAAYAAELPRSHPCAAAIAPGPGPLHILEPKA...HILEAERRARDGSPAHAM
Myotis lucifugus         PR..AREPRGSYSQEPQAGCSCASASAGEGPLHIHEPKA...HVTEAERRGREGSPVHVL
Equus caballus           LR..AREPRGGYSQDLQPSYACGSVAAVAGPLHILEPKS...HVTETEKRVQDSSPVHML
Xenopus laevis           PLFYDRAQLRTVMETYPDELEPIDTYSSAPANHLHIYEHKKQEEVEKEMRLKGVSPVQFS
Latimeria chalumnae      DDQVQLLPTTRQYPNEPLPPYPYASGRGVNISHHEMEQKTAAMGAEKDKRMKLFSTLHLM
Danio rerio              ..HTPQSQDAVSGPEHPMTSLGRSLGD..PPAYPFSHSMETVLTSPPAYQAHSIMQKAED
Electrophorus electricus ..HTPRSQDAVACPDQTMAVLGHQLMDTPPPAYHFAPSLDTVLASPLSFKPQPGIEEQEE

Homo sapiens
                                 790       800       810       820       830
Homo sapiens             AEAQRPWR.DGLLMPSMSESDLTTS.GRYRARRDSLKKRPVSDLLSGKKNIVEGLPPLGG
Mus musculus             VETHRPRR.DGLLTPSMSESDLTTS.GRYRARRDSVKKRPVSDLLSGKKSAVEGLPPLGG
Cavia porcellus          VEAQRPRR.EGLLTPSMSESDLSTS.GRYRARRDSLKKRPVSDLLSGKKNVVEGLPPLGG
Myotis lucifugus         AEAQRPRR.DGLLTPSMSESDLTTS.GRYRARRDSLKKRPVSDLLSGKKNIVEGLPPLGG
Equus caballus           VEAPRPRR.DGLLTPSMSESDLTTS.GRYRARRDSLKKRPVSDLLSGKKHIVEGLPPLGG
Xenopus laevis           GDFQPNRRGEGNIPPSASESDLTAVSIRYRPKKDQVKKRPVSDVLSGKKDIVEGLPPSGG
Latimeria chalumnae      GESRAQRKAEESLAPSMSESDLSTA.GRSRARRESTKNRPVPDVPIG.RSAVQGLPPLAG
Danio rerio              VGPLPPLREPGLMMPSVSEGDLSG....RLRKNRDISKRPVSDVPPGRRNIDG..LPPAG
Electrophorus electricus AGPLYLLGEAGALMPSVSEGNLSSQ.GRVKTKRELIKKRPVSDVPSARRNLDG..LPPPG

                                                                         α1'          α2'
Homo sapiens                                                      ooooooooooo   ooo
                          840       850       860       870       880       890
Homo sapiens             MKK.TRVDAKKIGPLKLAALNGLSLSRVPLPDEGKEVATRATNDERCKILEQRLEQGMVF
Mus musculus             MKK.TRADAKKIGPLKLAALNGLSLSRLPLPDEGKEVSTRATNDERCKVLEQRLEQGMVF
Cavia porcellus          MKK.TRVDAKKVGPLKLAALNGLSLSRLPLPDEGKEVSTRATNDERCKMLEQRLEQGMVF
Myotis lucifugus         MKK.SRVDAKKIGPLKLAALNGLSLSRLPLPDEGKDVSARATNDERCKILEQRLEQGMVF
Equus caballus           MKK.SRVDAKKIGPLKLAALNGLSLSRLPLPDEGNEVPTRATNDERCKILEQRLEQGMVF
Xenopus laevis           IKKGPRMEVKKIGPLKMAAWNGLSLSRLPLPDEEKEEPSRASNDDRCKLLEQRLEQGMVF
Latimeria chalumnae      IKKSGKPDMKKMGPLKLAVLNGLTLSRMPLPDEGREEPAKASNDERCKLLEQQLEQGMVF
Danio rerio              MKK.GRSEVKKVGPLKLAALNGLTLSRIPLPDDGKDDPESASNDERCKFLEQRLDQGMVF
Electrophorus electricus MKKSTRPEVKKMGPPKVAVLNGLTPTRVPTLGEGKDEPGKASNDERCKVLEQCLEQGMVF

                                            α1                                β1
Homo sapiens             oooo            ooooo                              ______
                         900       910       920       930       940       950
Homo sapiens             TEYERILKKRLVDGECSTARLPENAERNRFQDVLPYDDVRVELVPTKENNTGYINASHIK
Mus musculus             TEYERILKKRLVDGECSTARLPENAERNRFQDVLPYDDARVELVPTKENNTGYINASHIK
Cavia porcellus          TEYERILKKRLVDGECSTARLPENAERNRFQDVLPYDDARVELVPTKENNTGYINASHIK
Myotis lucifugus         TEYERILKKRLVDGECSTARLPENAERNRFQDVLPYDDARVELVPTKENNTGYINASHIK
Equus caballus           TEYERILKKRLVDGECSTARLPENAERNRFQDVLPYDDARVELVPTKENNTGYINASHIK
Xenopus laevis           TEYEQIGKKRLPDSECSIARLPENAERNRYQDVLPYDDSRVELVPTKENNTGYINASHIK
Latimeria chalumnae      TEYEQVPKKRAN.AECTIAQLPENAERNRFQDVLPYDDTRVELVPTKENNTGYINASHIK
Danio rerio              TEYEQVPK.KRPNSEYSIAQMPENDRNRFQDVLPYDDTRVELVPTKENNTGYINASHIR
Electrophorus electricus TEYEQVPK.RRPDGECSIAQLPENAERNRFQDVLPYDDSRVELVPTKENNTGYINASHIK

                               β2                α2            β3
Homo sapiens             -->  ------->       oooooooooo     --->
                          960       970       980       990      1000      1010
Homo sapiens             VSVSGIEWDYIATQGPLQNTCQDFWQMVWEQGIAIIAMVTAEEEGGREKSFRYWPRLGSR
Mus musculus             VSVSGIEWDYIATQGPLQNTCQDFWQMVWEQGVAIIAMVTAEEEGGREKSFRYWPRLGSR
Cavia porcellus          VSVGGIEWDYIATQGPLQNTCQDFWQMVWEQGIAIIAMVTAEEEGGREKSFRYWPRLGSR
Myotis lucifugus         VSVSGMEWDYIATQGPLQNTCQDFWQMVWEQGIAIIAMVTAEEEGGREKSFRYWPRLGSR
Equus caballus           VSVSGIEWDYIATQGPLQNTCQDFWQMVWEQGVAIIAMVTAEEEGGREKSFRYWPRLGSR
Xenopus laevis           MTVGGVEWDYIATQGPLQSTCQDFWQMVWEQGIAIIAMVTLDEEGGREKSYRYWPRLGSR
Latimeria chalumnae      VTVGGEEWDYIATQGPLQNTCQNFWQMVWEQGVTIIAMVTSEEEGGKEKCFRYWPWLGSR
Danio rerio              VTVRGKEWSYIASQGPLSSTCQDFWQMVWEQGVSIIAMVTAEEEGGREKSFRYWPRLGSR
Electrophorus electricus VTVVGEEWSYIASQGPLSNTCQDFWQMVWEQGVAIIAMVTAEEEGGREKSFRYWPRLGSR
```

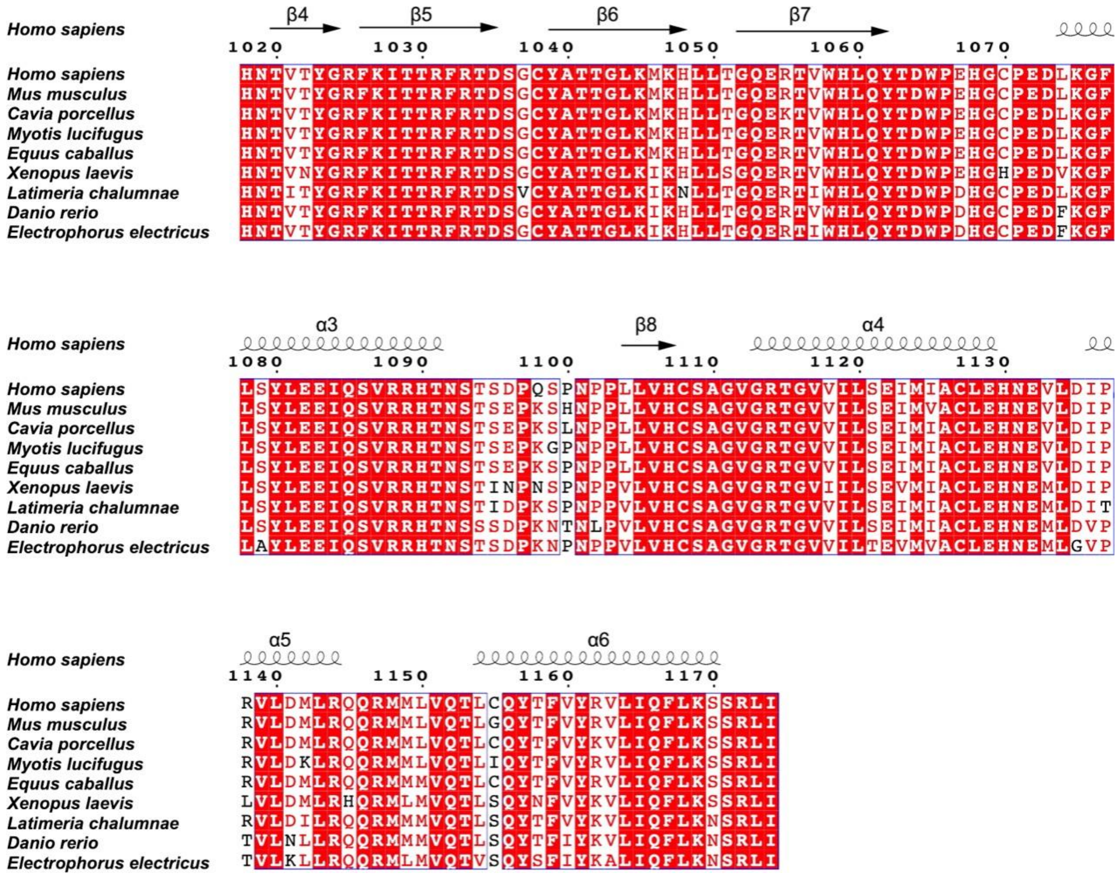

Fig. S1. Sequence alignment of human PTPN21 with the eight homologs from *Mus musculus, Cavia porcellus, Myotis lucifugus, Equus caballus, Xenopus laevis, Latimeria chalumnae, Danio rerio and Electrophorus electricus.*

Multiple sequence alignment of PTPN21 proteins was performed with Clustal Omega (https://www.ebi.ac.uk/Tools/msa/clustalo), and the resultant output was used as input files for the program ESPript (https://espript.ibcp.fr/ESPript/cgi-bin/ESPript.cgi).

Identical residues are white letters in red background; similar residues are red letters in white background; varied residues are dark letters.

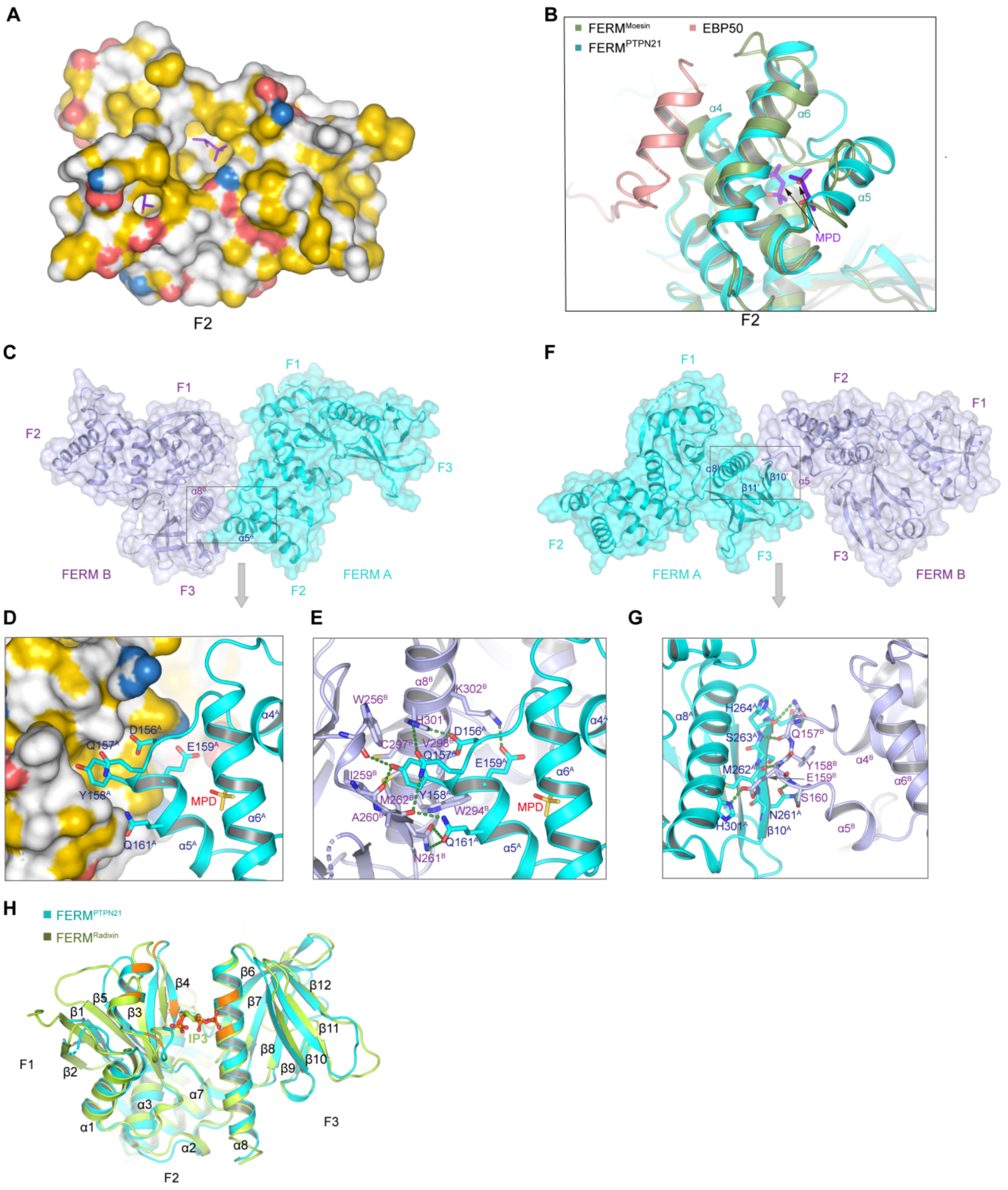

Fig. S2. Crystal structure of PTPN21 FERM domain.

(**A**) Surface representation of FERM^{PTPN21} F2 lobe with MPD bound, colored using the YRB color scheme, with hydrophobic, positively and negatively charged atoms in yellow, blue and red, respectively, other atoms in white. MPD molecules are shown as purple sticks.

(**B**) Superposition of FERM^{PTPN21} (cyan) and FERM^{moesin} (green)-EBP50 (red) complex (PDB: 1SGH). The MPD molecules in FERM^{PTPN21} are shown as purple sticks. EBP50 and MPD bind to different locations in FERM F2 lobe.

(**C** to **G**) The MPD-bound FERM domain (FERM A, cyan) and the apo FERM domain (FERM B, light-blue) form two kinds of contacts (**C** and **F**). Both interfaces involve residues surrounding Y158 (**D**, **E** and **G**), which adopts different conformations in FERM A and FERM B.

(**H**) Superposition of FERM^{PTPN21} (cyan) on FERM^{radixin} (PDB: 1J19, green). The IP3 molecule in FERM^{radixin} is shown as ball and sticks.

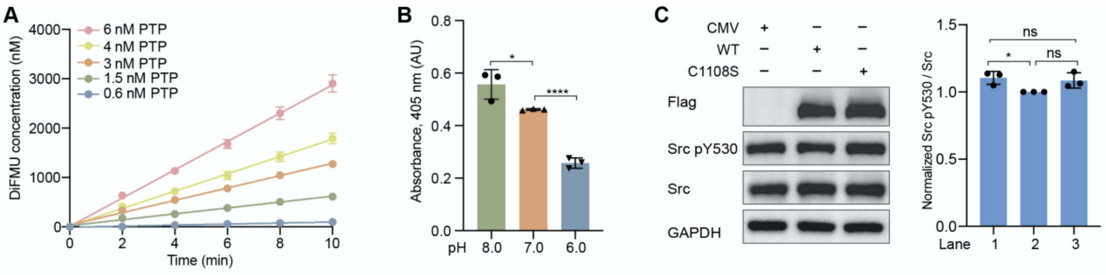

Fig. S3. Phosphatase activity of the PTP domain.

(**A**) Enzyme concentration titration curve. PTP was incubated with 40 µM DIFMUP at the desired concentrations varying from 0.6 to 6 nM.

(**B**) Phosphatase activity of PTP (17.7 µM) at different pH using *p*NPP (46.8 mM) as a substrate was monitored by absorbance at 405 nm.

(**C**) Immunoblot analysis of Src pY530 dephosphorylation by PTPN21 in HEK293T cells. HEK293T cells were transiently transfected with empty vector (CMV), N-terminal Flag-tagged-PTPN21 (WT) or PTPN21 C1108S (C1108S) vector for 48 h. Lysates were then collected for immunoblot analysis via sequentially probing with the indicated antibodies from left to right.

For all panels, error bars represent ± SEM of n = 3 independent experiments. In (**C**), the relative pSrc Y530 / Src values were calculated by normalizing to PTPN21 WT. In (**B** and **C**), Statistical analysis was performed using Student's t test (* $P < 0.05$, ns $P > 0.05$)

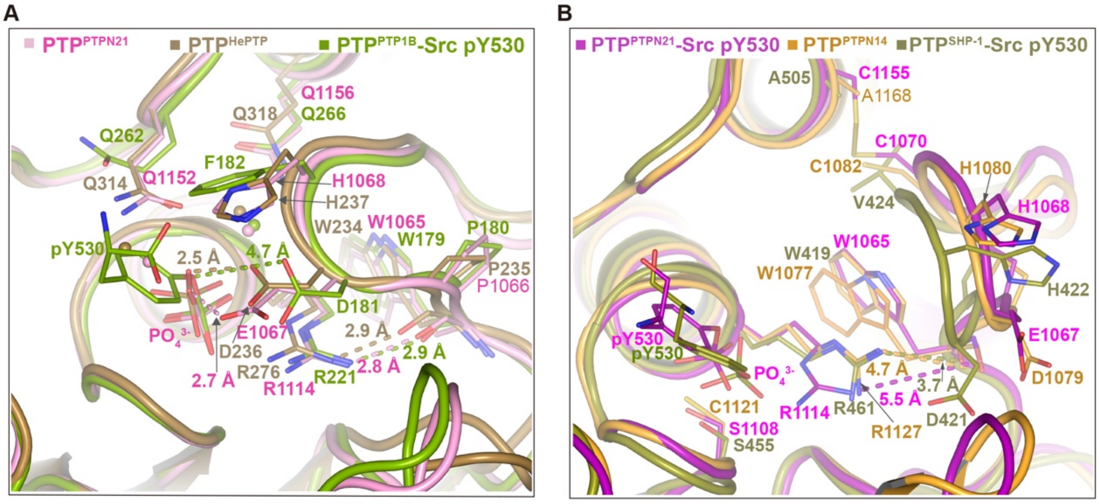

C

Kinetic parameters for hydrolysis of DiFMUP by PTP and mutants

	K_{cat} (s^{-1})	K_m (μM)	K_{cat}/K_m × 10^4 ($M^{-1} s^{-1}$)
PTP	1.23 ± 0.13	28.57 ± 3.74	4.32 ± 0.22
PTP C1070S	0.93 ± 0.10	24.77 ± 1.66	3.79 ± 0.53
PTP C1155S	1.07 ± 0.08	22.75 ± 2.30	4.70 ± 0.27
PTP Q1152A	0.13 ± 0.01	10.45 ± 0.57	1.28 ± 0.06
PTP Q1156A	0.30 ± 0.03	18.45 ± 1.77	1.63 ± 0.10
PTP R1114Q	N.D	N.D	N.D

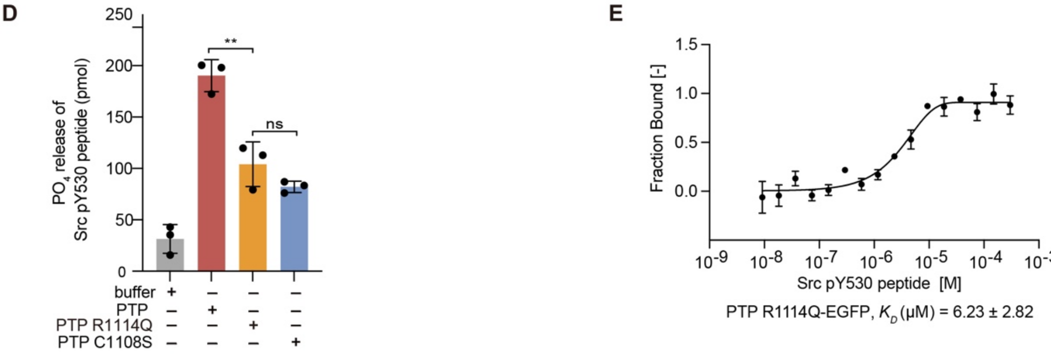

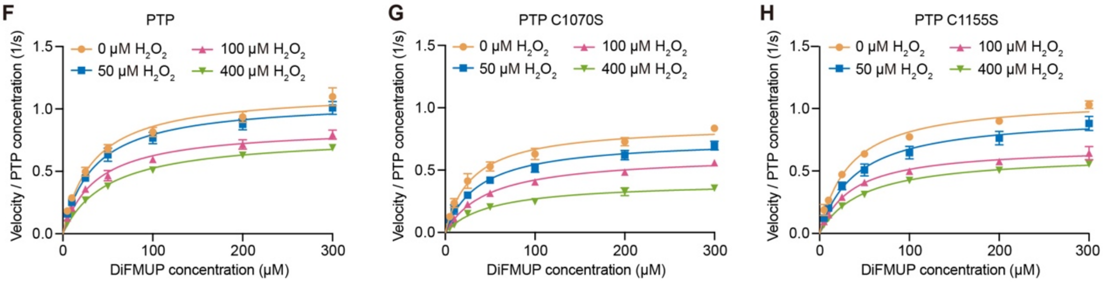

Kinetic parameters for hydrolysis of DiFMUP by PTP, PTP C1070S and PTP C1155S in the presence of different concentration of H_2O_2

H_2O_2 / Enzyme	0 μM			50 μM			100 μM			400 μM		
	K_{cat} (s^{-1})	K_m (μM)	K_{cat}/K_m × 10^4($M^{-1}s^{-1}$)	K_{cat} (s^{-1})	K_m (μM)	K_{cat}/K_m × 10^4 ($M^{-1}s^{-1}$)	K_{cat} (s^{-1})	K_m (μM)	K_{cat}/K_m × 10^4 ($M^{-1}s^{-1}$)	K_{cat} (s^{-1})	K_m (μM)	K_{cat}/K_m × 10^4 ($M^{-1}s^{-1}$)
PTP	1.20 ± 0.05	36.49 ± 4.73	3.33 ± 0.29	1.07 ± 0.03	34.67 ± 0.21	3.10 ± 0.11	0.87 ± 0.02	38.20 ± 1.37	2.29 ± 0.12	0.78 ± 0.04	45.62 ± 5.14	1.73 ± 0.27
PTP C1070S	0.87 ± 0.02	34.43 ± 4.62	2.57 ± 0.28	0.77 ± 0.04	41.80 ± 2.85	1.84 ± 0.13	0.52 ± 0.07	44.55 ± 2.74	1.18 ± 0.12	0.43 ± 0.04	59.54 ± 6.89	0.73 ± 0.06
PTP C1155S	1.11 ± 0.04	35.20 ± 2.76	3.16 ± 0.14	0.98 ± 0.03	37.71 ± 2.74	2.61 ± 0.20	0.70 ± 0.02	36.55 ± 2.47	1.91 ± 0.18	0.66 ± 0.02	46.94 ± 1.11	1.40 ± 0.07

Fig. S4. Crystal structure of the PTP domain.

(**A**) Superposition of PTP^PTPN21 (pink), PTP^HePTP (PDB: 1ZC0, brown) and PTP^PTP1B-Src pY530 complex (PDB: 1PTV, green), the WPD/E loops in them are in a closed stated. The key residues are shown as sticks. The distances between residues are indicated by dashed lines (pink for PTP^PTPN21, orange for PTP^HePTP and green for PTP^PTP1B-Src pY530). Spheres denote water molecules.

(**B**) Superposition of PTP^{PTPN21}-Src pY530 complex (magenta), PTP^{PTPN14} (PDB: 2BZL, orange) and $PTP^{SHP\text{-}1}$-Src pY530 complex (PDB: 1FPR, olive), the WPD/E loop are in the open state. The distances between residues from PTP^{PTPN21}, PTP^{PTPN14} and $PTP^{SHP\text{-}1}$ are indicated with magenta, orange and olive dashed lines, respectively. Spheres denote water molecules.
(**C**) Kinetic parameters of PTP, PTP C1070S, PTP C1155S, PTP Q1152A, PTP Q1156A and PTP R1114Q using DiFMUP as a substrate.
(**D**) Phosphatase activity of PTP, PTP C1108S and PTP R1114Q using Src pY530 peptide (STEPQpYQPGENL) as a substrate, the released phosphate was detected by malachite reagents. The assays were performed by incubating 2.68 μM protein with 268 μM Src pY530 peptide for 30 min at 30°C.
(**E**) Microscale thermophoresis (MST) binding affinity measurement of Src pY530 peptide with PTP R1114Q. Fluorescent PTP R1114Q-EGFP (80 nM) was mixed with an increasing amount of Src pY530 peptide. K_D, dissociation constant.
(**F** to **H**) Michaelis-Menten plots of initial rate vs. substrate (DiFMUP) concentration using 5 nM PTP (**F**), PTP C1070S (**G**) and PTP C1155S (**H**) after incubating with various concentration of H_2O_2. The resulting enzyme kinetic parameters were summarized in the table.
(**A** to **F**) Error bars represent ± SEM of n = 3 independent experiments.

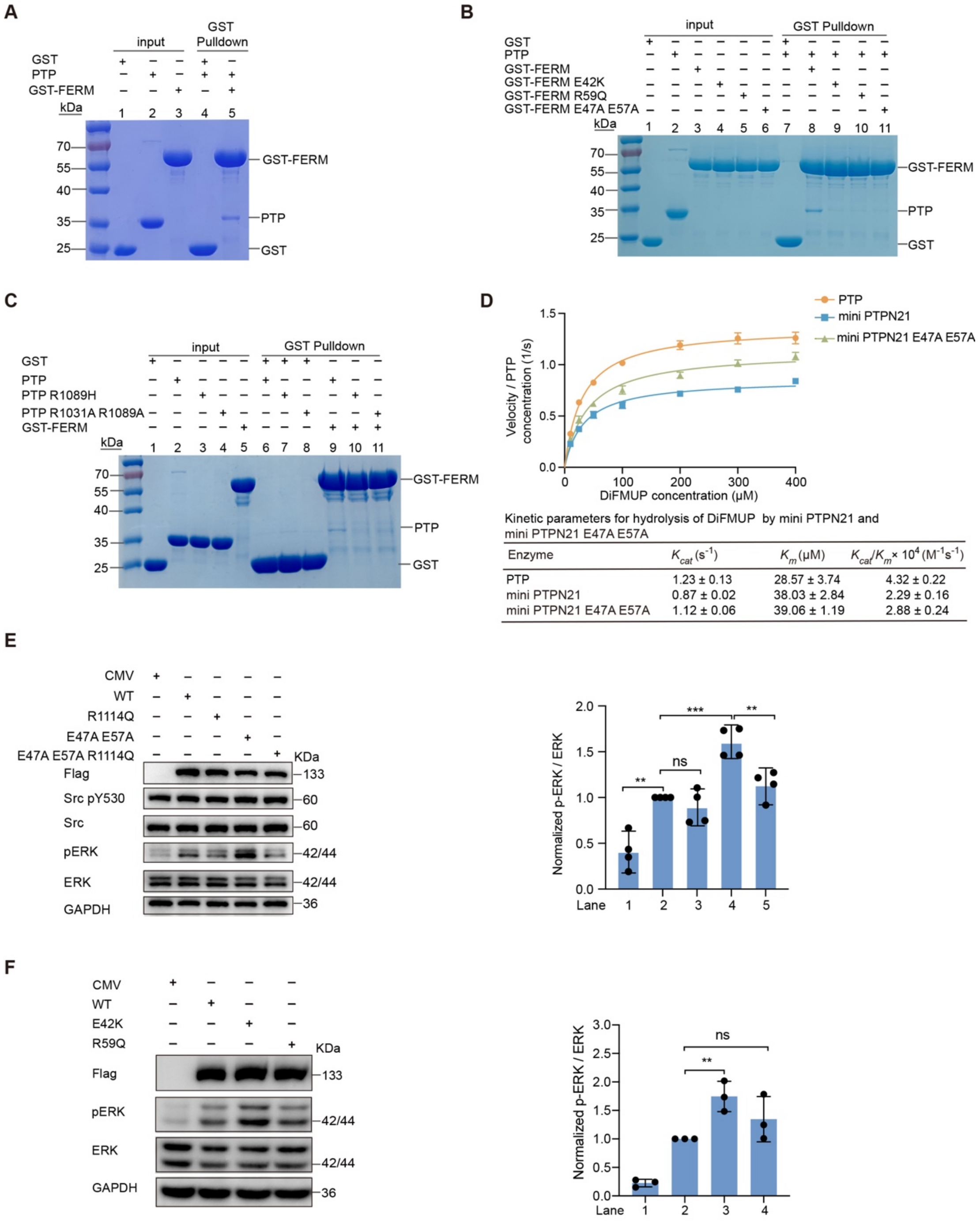

Kinetic parameters for hydrolysis of DiFMUP by mini PTPN21 and mini PTPN21 E47A E57A

Enzyme	K_{cat} (s^{-1})	K_m (μM)	$K_{cat}/K_m \times 10^4$ ($M^{-1}s^{-1}$)
PTP	1.23 ± 0.13	28.57 ± 3.74	4.32 ± 0.22
mini PTPN21	0.87 ± 0.02	38.03 ± 2.84	2.29 ± 0.16
mini PTPN21 E47A E57A	1.12 ± 0.06	39.06 ± 1.19	2.88 ± 0.24

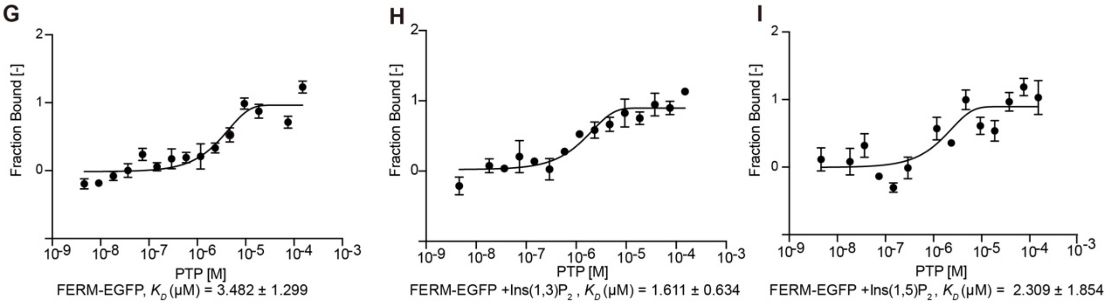

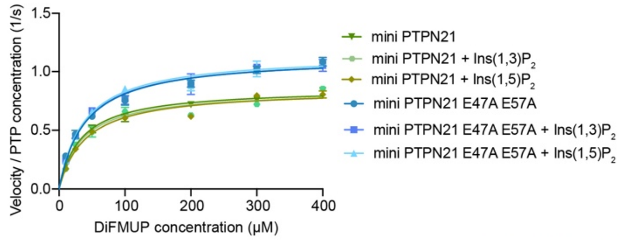

Kinetic parameters for hydrolysis of DiFMUP by mini PTPN21 and mini PTPN21 E47A E57A in the presence of Ins(1,3)P_2 and Ins(1,5)P_2

Enzyme	K_{cat} (s^{-1})	K_m (μM)	K_{cat}/K_m × 10^4 ($M^{-1}s^{-1}$)
mini PTPN21	0.87 ± 0.02	38.03 ± 2.84	2.29 ± 0.16
mini PTPN21+Ins(1,3)P_2	0.85 ± 0.04	38.03 ± 2.00	2.24 ± 0.13
mini PTPN21+Ins(1,5)P_2	0.86 ± 0.01	39.86 ± 0.05	2.17 ± 0.03
mini PTPN21 E47A E57A	1.12 ± 0.06	39.06 ± 1.19	2.88 ± 0.24
mini PTPN21 E47A E57A+Ins(1,3)P_2	1.13 ± 0.01	38.07 ± 0.61	2.96 ± 0.04
mini PTPN21 E47A E57A+Ins(1,5)P_2	1.16 ± 0.02	39.76 ± 1.58	2.93 ± 0.06

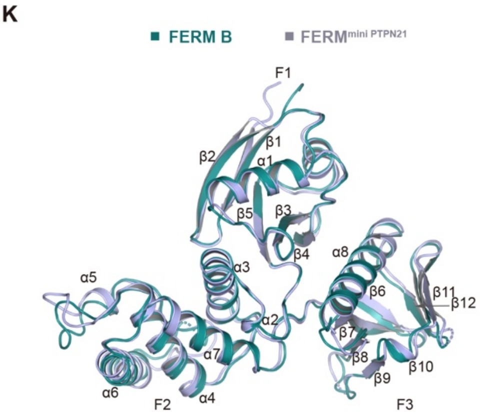

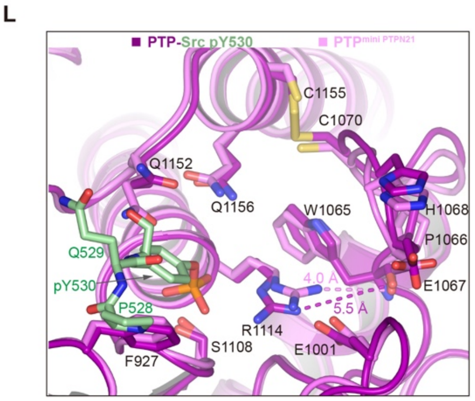

Fig. S5. PTPN21 FERM domain interacts with the PTP domain and inhibits PTP activity.

(**A**) GST pulldown assay showed that PTP^{PTPN21} domain could associate with $FERM^{PTPN21}$ domain.

(**B**) GST pulldown assay showed that cancer-related mutations of PTPN21 FERM (FERM E42K, FERM R59Q) abrogated the binding between PTPN21 FERM and PTP domains.

(**C**) GST pulldown assay showed that cancer-related mutations of PTP (PTP R1089H) abrogated the binding between PTPN21 FERM and PTP domains.

(D) Michaelis-Menten plots of initial rate vs. substrate (DiFMUP) concentration using 5 nM PTP, mini PTPN21 (FERM-PTP fusion) and mini PTPN21 with FERM E47A E57A mutation (mini PTPN21 E47A E57A)**.** The resulting enzyme kinetic parameters were summarized in the table. Error bars represent ± SEM of n = 3 independent experiments.

(E) PTPN21 with R1114Q or E47A E57A R1114Q mutation had little effect on ERK activation. HEK293T cells were transiently transfected with empty vector (CMV), N-terminal Flag-tagged PTPN21 (WT), PTPN21 R1114Q (R1114Q), PTPN21 E47A E57A (E47A E57A) or PTPN21 E47A E57A R1114Q (E47A E57A R1114Q) vector for 48 h. Lysates were then collected for immunoblot

analysis via sequentially probing with the indicated antibodies. For quantitative analysis, each lane of western blots was sequentially shown as lane 1, lane 2, lane 3, lane 4 and lane 5 from left to right.
(F) PTPN21 with E42K mutation resulted in more ERK activation. HEK293T cells were transiently transfected with empty vector (CMV), N-terminal Flag-tagged PTPN21 (WT), PTPN21 E42K (E42K) or PTPN21 R59Q (R59Q) vector for 48 h. Lysates were then collected for immunoblot analysis via sequentially probing with the indicated antibodies. For quantitative analysis, each lane of western blots was sequentially shown as lane 1, lane 2, lane 3 and lane 4 from left to right.
(G) MST titration results of FERM-EGFP with PTP. Fluorescent FERM-EGFP (80 nM) was mixed with an increasing amount of PTP. K_D, dissociation constant. Data represent mean ± SEM from n = 3 independent experiments.
(H and **I)** MST titration results of FERM-EGFP with PTP in the presence of Ins(1,3)P_2 (**H**) or Ins(1,5)P_2 (**I**). 1 μM Ins(1,3)P_2 or Ins(1,5)P_2 was mixed with fluorescent FERM-EGFP (80 nM) and then titrated with an increasing concentration of PTP. K_D, dissociation constant. Data represent mean ± SEM from n = 3 independent experiments.
(J) Michaelis-Menten plots of initial rate vs. substrate (DiFMUP) concentration using 5 nM mini PTPN21 (FERM-PTP fusion) and mini PTPN21 with FERM E47A E57A mutation (mini PTPN21 E47A E57A) in the presence of Ins(1,3)P_2 and Ins(1,5)P_2**.** The resulting enzyme kinetic parameters were summarized in the table. Error bars represent ± SEM of n = 3 independent experiments.
(**K**) Superposition of FERM B (light-blue) with the FERM domain from the mini PTPN21 (teal). The secondary structural elements are labeled.
(**L**) Superposition of the PTP (magenta)-Src pY530 peptide (green) complex with PTP (pink) from the mini PTPN21. The WPD/E loops in both structures are in the open state. Dashed lines indicate the distance between R1114 guanidino group with W1065 carbonyl oxygen.
(**E** to **G**) Error bars represent ± SEM of n ≥ 3 independent experiments. The relative values were calculated by normalizing to PTPN21 WT. Statistical analysis was performed using a Student's t test (*** $P < 0.001$, ** $P < 0.01$, * $P < 0.05$, ns $P > 0.05$).

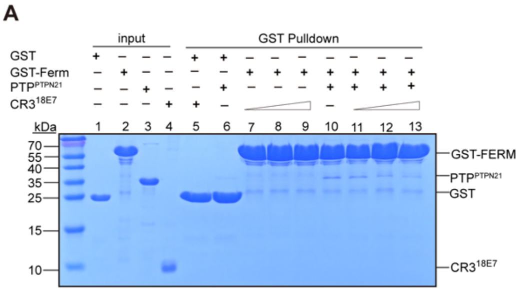

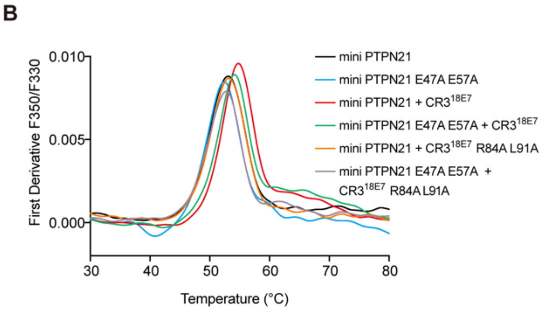

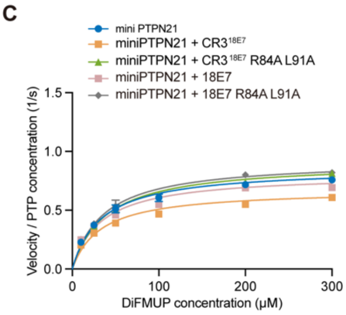

Kinetic parameters for hydrolysis of DiFMUP by mini PTPN21 fusion in the presence of CR3^{18E7},18E7 and mutants

Enzyme	K_{cat} (s^{-1})	K_m (μM)	K_{cat}/K_m × 10^4 (M^{-1} s^{-1})
mini PTPN21	0.87 ± 0.01	38.03 ± 2.84	2.29 ± 0.16
mini PTPN21 + CR3^{18E7}	0.72 ± 0.03	34.69 ± 1.42	2.08 ± 0.09
mini PTPN21 + CR3^{18E7} R84A L91A	0.93 ± 0.06	38.97 ± 3.61	2.42 ± 0.27
mini PTPN21 + 18E7	0.79 ± 0.01	35.19 ± 1.32	2.26 ± 0.11
mini PTPN21 + 18E7 R84A L91A	0.90 ± 0.02	39.86 ± 0.73	2.25 ± 0.08

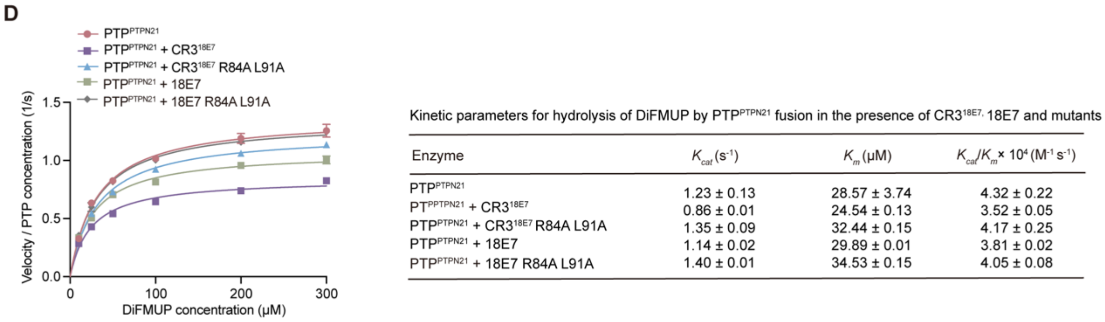

Kinetic parameters for hydrolysis of DiFMUP by PTP^{PTPN21} fusion in the presence of CR3^{18E7,} 18E7 and mutants

Enzyme	K_{cat} (s^{-1})	K_m (μM)	K_{cat}/K_m × 10^4 (M^{-1} s^{-1})
PTP^{PTPN21}	1.23 ± 0.13	28.57 ± 3.74	4.32 ± 0.22
PT^{PPTPN21} + CR3^{18E7}	0.86 ± 0.01	24.54 ± 0.13	3.52 ± 0.05
PTP^{PTPN21} + CR3^{18E7} R84A L91A	1.35 ± 0.09	32.44 ± 0.15	4.17 ± 0.25
PTP^{PTPN21} + 18E7	1.14 ± 0.02	29.89 ± 0.01	3.81 ± 0.02
PTP^{PTPN21} + 18E7 R84A L91A	1.40 ± 0.01	34.53 ± 0.15	4.05 ± 0.08

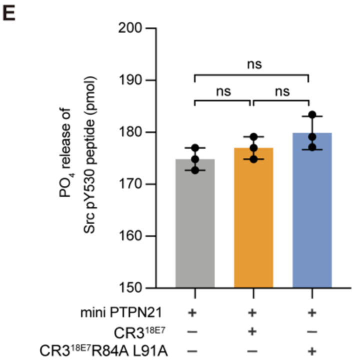

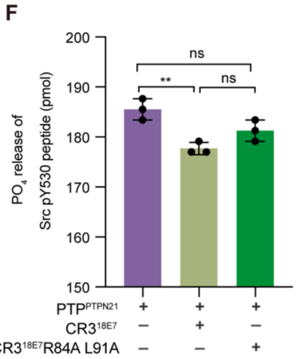

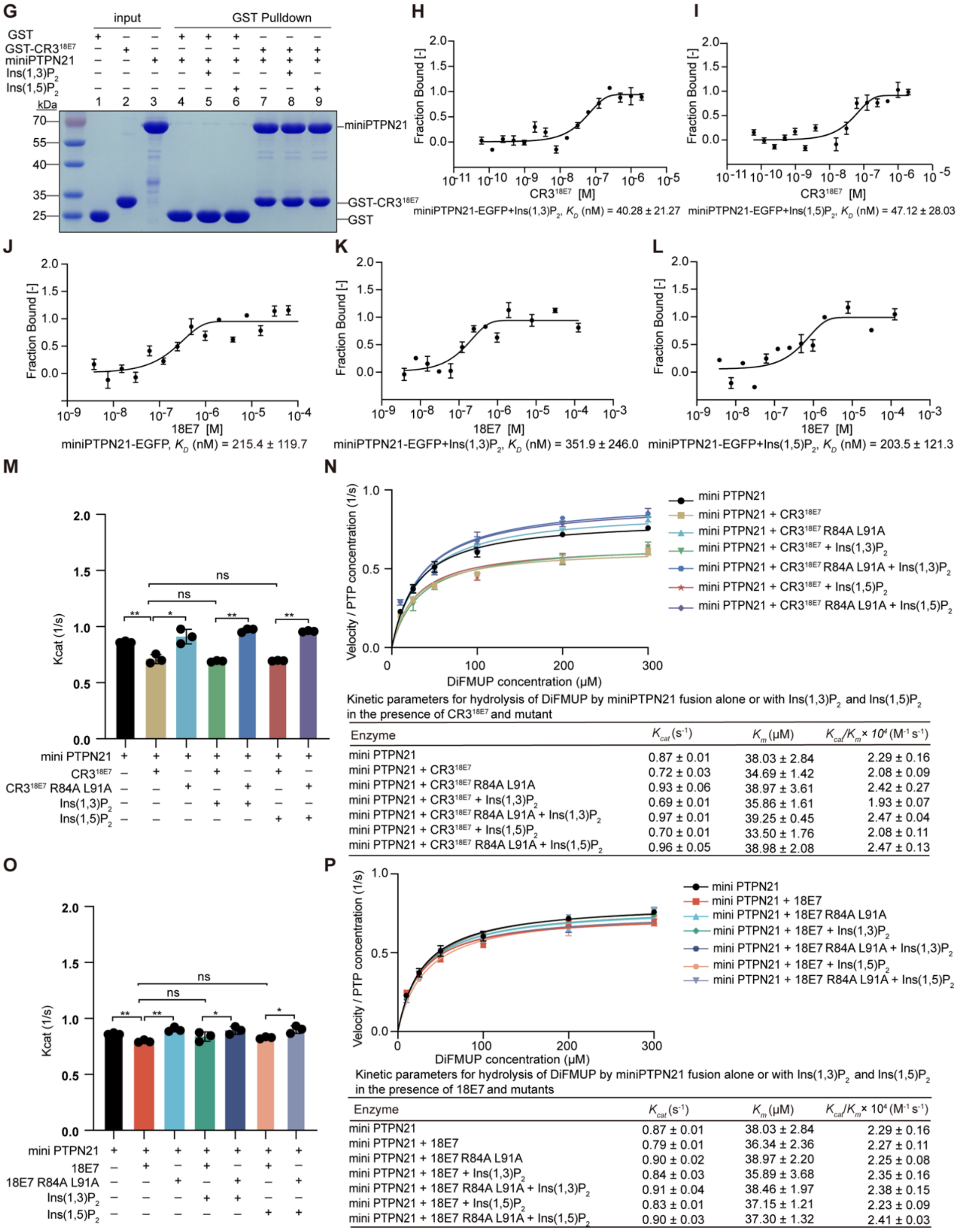

Kinetic parameters for hydrolysis of DiFMUP by miniPTPN21 fusion alone or with Ins(1,3)P_2 and Ins(1,5)P_2 in the presence of CR3^{18E7} and mutant

Enzyme	K_{cat} (s^{-1})	K_m (μM)	$K_{cat}/K_m \times 10^4$ ($M^{-1} s^{-1}$)
mini PTPN21	0.87 ± 0.01	38.03 ± 2.84	2.29 ± 0.16
mini PTPN21 + CR3^{18E7}	0.72 ± 0.03	34.69 ± 1.42	2.08 ± 0.09
mini PTPN21 + CR3^{18E7} R84A L91A	0.93 ± 0.06	38.97 ± 3.61	2.42 ± 0.27
mini PTPN21 + CR3^{18E7} + Ins(1,3)P_2	0.69 ± 0.01	35.86 ± 1.61	1.93 ± 0.07
mini PTPN21 + CR3^{18E7} R84A L91A + Ins(1,3)P_2	0.97 ± 0.01	39.25 ± 0.45	2.47 ± 0.04
mini PTPN21 + CR3^{18E7} + Ins(1,5)P_2	0.70 ± 0.01	33.50 ± 1.76	2.08 ± 0.11
mini PTPN21 + CR3^{18E7} R84A L91A + Ins(1,5)P_2	0.96 ± 0.05	38.98 ± 2.08	2.47 ± 0.13

Kinetic parameters for hydrolysis of DiFMUP by miniPTPN21 fusion alone or with Ins(1,3)P_2 and Ins(1,5)P_2 in the presence of 18E7 and mutants

Enzyme	K_{cat} (s^{-1})	K_m (μM)	$K_{cat}/K_m \times 10^4$ ($M^{-1} s^{-1}$)
mini PTPN21	0.87 ± 0.01	38.03 ± 2.84	2.29 ± 0.16
mini PTPN21 + 18E7	0.79 ± 0.01	36.34 ± 2.36	2.27 ± 0.11
mini PTPN21 + 18E7 R84A L91A	0.90 ± 0.02	38.97 ± 2.20	2.25 ± 0.08
mini PTPN21 + 18E7 + Ins(1,3)P_2	0.84 ± 0.03	35.89 ± 3.68	2.35 ± 0.16
mini PTPN21 + 18E7 R84A L91A + Ins(1,3)P_2	0.91 ± 0.04	38.46 ± 1.97	2.38 ± 0.15
mini PTPN21 + 18E7 + Ins(1,5)P_2	0.83 ± 0.01	37.15 ± 1.21	2.23 ± 0.09
mini PTPN21 + 18E7 R84A L91A + Ins(1,5)P_2	0.90 ± 0.03	37.30 ± 1.32	2.41 ± 0.03

Fig. S6. HPV18E7 binds to and regulates PTPN21.

(**A**) GST pulldown assay with PTP and GST-FERM. Addition of 20 μM, 40 μM, and 80 μM CR3^{18E7} reduced the interaction between GST-FERM (40 μM) and PTP (40 μM) to different degrees. The volume for each reaction was 50 μL, 15 μL was loaded onto the gel; for input, 4 μL was loaded.

(**B**) Thermal stabilities of mini PTPN21 alone or in the presence of 5 nM CR3^{18E7} or CR3^{18E7} R84A L91A mutant measured by nano-DSF. Melting curves are shown, related to Fig. 5F in the main text. Data were from two independent experiments.

(**C**) Enzyme kinetic parameters for the phosphatase activity of 5nM mini PTPN21 alone and in the

presence of 5 nM CR3^{18E7}, CR3^{18E7} R84A L91A mutant, 18E7 or 18E7 R84A L91A mutant, related to Fig. 5I in the main text. Error bars represent ± SEM of n = 3 independent experiments.

(**D**) Enzyme kinetic parameters for the phosphatase activity of PTP alone or in the presence of 5 nM CR3^{18E7}, CR3^{18E7} R84A L91A mutant, 18E7 or 18E7 R84A L91A mutant, related to Figure 5J in the main text. Error bars represent ± SEM of n = 3 independent experiments.

(E) Phosphatase activity of mini PTPN21 alone or in the presence of CR3^{18E7}or CR3^{18E7} R84A L91A mutant using Src pY530 peptide (STEPQpYQPGENL) as a substrate, the released phosphate was detected by malachite reagents. The assays were performed by incubating 2.68 μM protein with 268 μM Src pY530 peptide for 30 min at 30°C. Statistical analysis was performed using Student's t test (ns $P > 0.05$).

(F) Phosphatase activity of PTP alone or in the presence of CR3^{18E7}, CR3^{18E7} R84A L91A mutant using Src pY530 peptide (STEPQpYQPGENL) as a substrate, the released phosphate was detected by malachite reagents. The assays were performed by incubating 2.68 μM protein with 268 μM Src pY530 peptide for 30 min at 30°C. Statistical analysis was performed using Student's t test (ns $P >$ 0.05).

(**G**) GST pulldown assay showed that addition of 40 μM Ins(1,3)P_2 or Ins(1,5)P_2 had little effect on the interaction between GST-CR3^{18E7} (40 μM) and mini PTPN21 (40 μM).

(**H** and **I**) MST titration results of mini PTPN21-EGFP with CR3^{18E7} in the presence of Ins(1,3)P_2 (**H**) and Ins(1,5)P_2 (**I**). 1 μM Ins(1,3)P_2 or Ins(1,5)P_2 was combined with fluorescent mini PTPN21-EGFP (80 nM) and then titrated with an increasing concentration of CR3^{18E7}. K_D, dissociation constant. Data represent mean ± SEM from n = 3 independent experiments.

(**J** to **L**) MST titration results of mini PTPN21-EGFP with 18E7 alone (**J**) or in the presence of Ins(1,3)P_2 (**K**) and Ins(1,5)P_2 (**L**). 1 μM Ins(1,3)P_2 or Ins(1,5)P_2 was combined with fluorescent mini PTPN21-EGFP (80 nM) and then titrated with an increasing concentration of 18E7. K_D, dissociation constant. Data represent mean ± SEM from n = 3 independent experiments.

(**M** and **N**) Michaelis-Menten plots of initial rate vs. substrate (DiFMUP) concentration using 5 nM mini PTPN21alone or with 1 μM Ins(1,3)P_2 and Ins(1,5)P_2 in the presence of 5 nM CR3^{18E7} and CR3^{18E7} R84A L91A mutant. The *Kcat* values were measured using DiFMUP substrate ranging from 10 to 300 μM (M). The resulting enzyme kinetic parameters were summarized in the table (**N**). Error bars represent ± SEM of n = 3 independent experiments.

(**O** and **P**) Michaelis-Menten plots of initial rate vs. substrate (DiFMUP) concentration using 5 nM mini PTPN21 alone or with 1 μM Ins(1,3)P_2 and Ins(1,5)P_2 in the presence of 5 nM 18E7 and 18E7 R84A L91A mutant**.** The *Kcat* values were measured using DiFMUP substrate ranging from 10 to 300 μM in (**O**). The resulting enzyme kinetic parameters were summarized in the table in (**P**). Error bars represent ± SEM of n = 3 independent experiments.

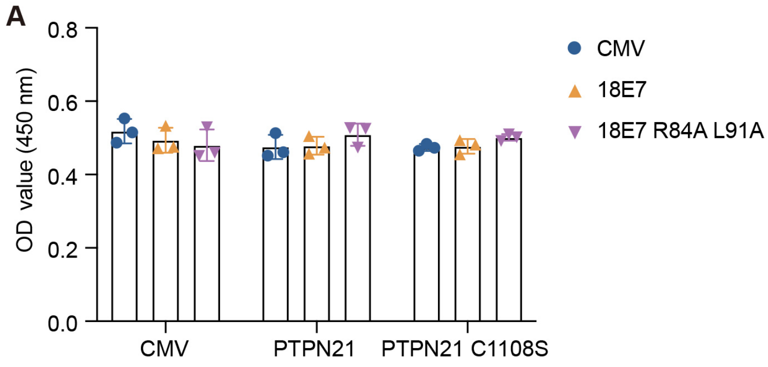

Fig. S7. 293T cell proliferation was not affected by overexpression of PTPN21 or 18E7.
(**A**) CCK-8 assay showing HEK293T cells transiently expressing 0.4 μg/μl PTPN21 or PTPN21 C1108S plasmid in combination with 0.1 μg/μl CMV: empty vector, 18E7 or 18E7 R84AL91A plasmid did not significantly affect cell proliferation after culturing for 48 h (n = 3). OD, optical density.

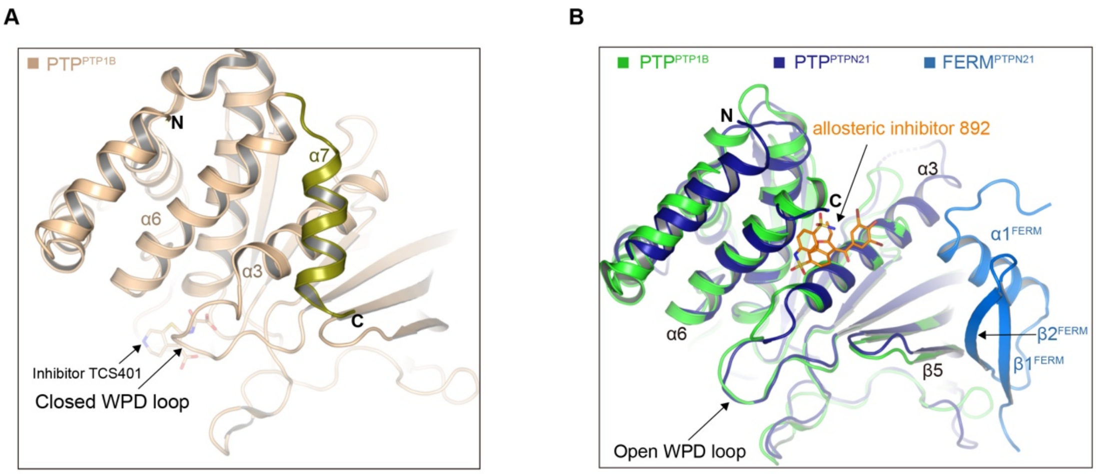

Fig. S8. Structural comparison of PTP^{PTPN21} with PTP^{PTP1B}.

(**A**) Structure of PTP1B (beige) in complex with the active site inhibitor TCS401 (PDB: 5K9W). The WPD loop is in the closed conformation. α7, α6 and α3 form the allosteric site. The α7 in PTP1B is highlighted with olive color, PTPN21 lacks this alpha helix.

(**B**) Superposition of PTP1B (green)-allosteric inhibitor 892 (orange) complex (PDB: 1T49) with PTP^{PTPN21} (blue) and FERM^{PTPN21} (marine) complex. For clarity, only β1, β2 and α1 from FERM^{PTPN21} are shown. The WPD loops are in the open conformation.

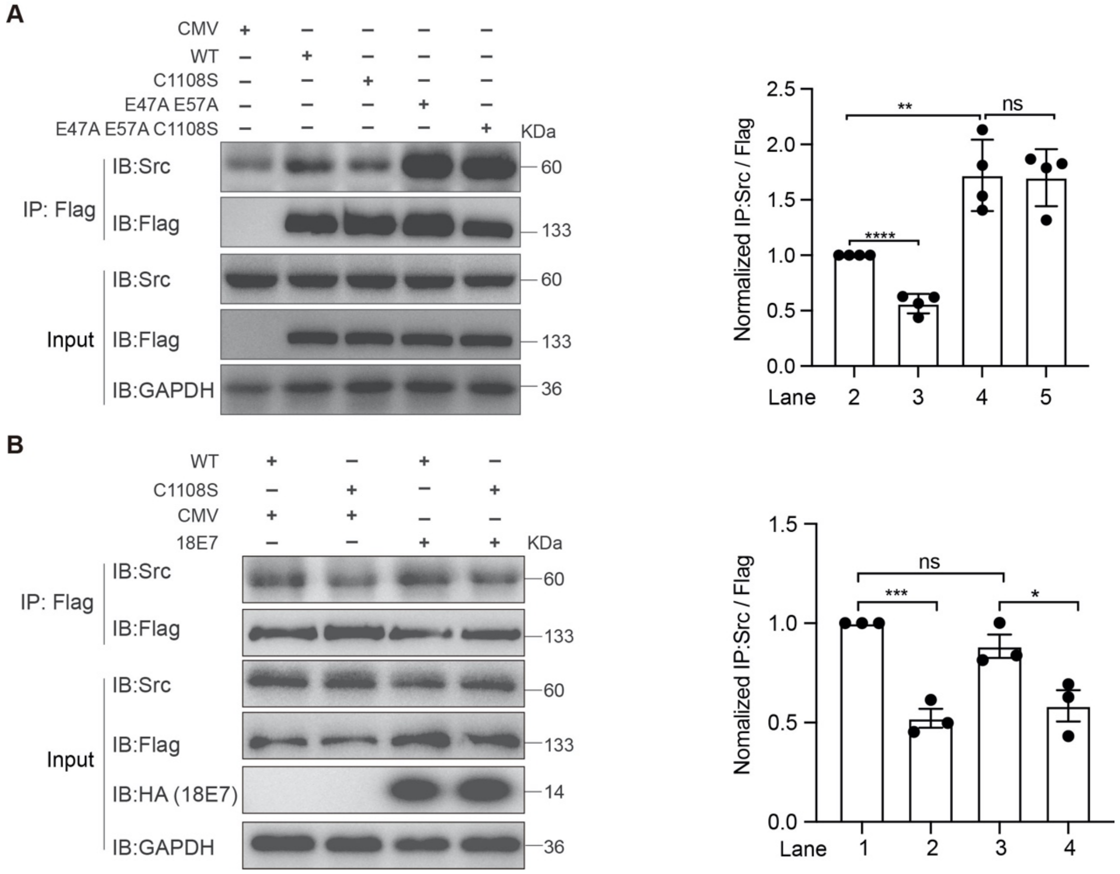

Fig. S9. Co-immunoprecipitation analysis of the interaction between PTPN21 and endogenous Src in HEK293T cells.

(**A**) PTPN21 with E47A E57A mutation pull-downed more Src than PTPN21 WT. WT or mutant PTPN21 were N-terminal Flag-tagged, CMV: empty vector, C1108S: PTPN21 C1108S, E47A E57A: PTPN21 E47A E57A, E47A E57A C1108S: PTPN21 E47A E57A C1108S. For quantitative analysis, each lane of western blots was sequentially shown as lane 2 to 5 from left to right.

(**B**) The interaction between PTPN21 and endogenous Src was not significantly affected in the presence of 18E7. CMV: empty vector. For quantitative analysis, each lane of western blots was sequentially shown as lane 1 to 4 from left to right.

(**A** and **B**) Error bars represent ± SEM of n ⩾ 3 independent experiments. The relative values were calculated by normalizing to PTPN21 WT. Statistical analysis was performed using Student's t test (**** $P < 0.0001$, *** $P < 0.001$, ** $P < 0.01$, * $P < 0.05$, ns $P > 0.05$).

Table S1. Data Collection and Refinement Statistics

Data Set	PTPN21 FERM	PTPN21 PTP	PTPN21 PTP-Src Y530	PTPN21 FERM-PTP
**Data collection**				
PDB code	8GVL	8GVV	8GWH	8GXE
Space group	*P 21 21 21*	*C 2*	*P 43 21 2*	*C 2 2 21*
a, b, c (Å)	41.452 105.443 162.302	80.382 56.433 72.099	104.545 104.545 71.868	72.313 228.246 117.05
α, β, γ (°)	90, 90, 90	90, 111.898, 90	90, 90, 90	90, 90, 90
Resolution (Å)	27.96 - 2.1 (2.175 - 2.1)	24.35 - 1.8 (1.864 - 1.8)	42.27 - 2.0 (2.071 - 2.0)	47.84 - 3.0 (3.107 - 3.0)
Observed reflections	305345 (29057)	184567 (16555)	391724 (39037)	263347 (26749)
Unique reflections	42547 (4222)	27851 (2738)	27377 (2656)	19796 (1918)
R_{merge} (%)	7.054 (54.1)	5.156 (35.4)	11.88 (92.86)	28.93 (158.3)
R_{pim} (%)	2.846 (22.16)	2.156 (15.61)	3.277 (24.78)	8.139 (43.33)
I/σ(I)	16.19 (3.18)	22.44 (4.67)	14.13 (3.36)	8.89 (1.67)
$CC_{1/2}$	0.998 (0.897)	0.999 (0.957)	0.993 (0.921)	0.994 (0.809)
Completeness (%)	99.85 (99.98)	99.85 (99.78)	99.63 (99.40)	99.69 (99.43)
Multiplicity	7.2 (6.9)	6.6 (6.0)	14.3 (14.7)	13.3 (13.9)
**Refinement**				
R_{work}/R_{free} (%)	19.58/22.60	19.28/22.09	18.79/20.88	22.17/26.66
No. protein atoms	573	286	299	568
No. ligand atoms	24	8	0	1
No. solvent atoms	237	242	142	16
Average B-factor ($Å^2$)	50.14	31.72	46.99	92.13
Protein B-factor ($Å^2$)	50.39	31.19	46.94	92.32
Solvent B-factor ($Å^2$)	45.98	36.97	47.96	41.73
Rmsd bond lengths (Å)	0.008	0.008	0.008	0.004
Rmsd bond angles (°)	0.89	1.02	0.99	0.70
Ramachandran outliers (%)	0.00	0.36	0.34	0.18
Ramachandran favored (%)	97.72	96.43	96.93	96.93

Values in parentheses are for the highest-resolution shell.
